# Research trends and frontiers in exercise for movement disorders: A bibliometric analysis of global research from 2010 to 2021

**DOI:** 10.3389/fnagi.2022.977100

**Published:** 2022-09-07

**Authors:** Ji-Wei Chen, Yue Guan, Yi-Li Zheng, Kun Zhu

**Affiliations:** ^1^School of Physical Education, Shanghai University of Sport, Shanghai, China; ^2^Shanghai Sports University Library, Shanghai University of Sport, Shanghai, China; ^3^Department of Sport Rehabilitation, Shanghai University of Sport, Shanghai, China

**Keywords:** exercise, non-pharmacological treatment, movement disorders, bibliometrics, visualization, frontier hotspots

## Abstract

**Objective:**

To conduct a bibliometric analysis of trends and frontiers on exercise-based non-pharmacological treatments for movement disorders published between 2010 and 2021.

**Methods:**

The Web of Science (WOS) Core Collection database was searched for articles published between 2010 and 2021. The CiteSpace software was used for in-depth analysis of the countries, institutions, journals, and collaboration networks among authors and their types of articles, developmental directions, references, and hot keywords of published articles.

**Results:**

A total of 2,626 published articles were retrieved by search formula and included in the analysis. The number of publications fluctuated during this period, with 96 countries, 3,058 institutions, and 886 academic journals having published articles in this area, with subject classifications that focused on Clinical Neurology and Neurosciences. The United States has maintained its dominant and most influential position in exercise-based non-pharmacological research on movement disorders. Among research institutions and journals, the League of European Research Universities and *Movement Disorders* journals published the highest number of academic articles. In the last five years, the hot research topics by burst keyword analysis, are focused on treatments, research advances, and clinical treatments.

**Conclusion:**

Research on exercise-based non-pharmacological treatments for movement disorders is generally on the rise from 2010 to 2021. The bibliometric analysis of this area will help provide potential collaborations among researchers, frontiers, and directions for development.

## Introduction

Movement disorders belong to a diverse group of neurological disorders that manifest as neurological disabilities represented by degeneration of motor function or motor dysfunction and other disorders related to motor control. In recent years, it has received increasing attention in the field related to movement disorders (Fox et al., 2018). Symptoms of movement disorders include non-subjective conscious movements: tics, chorea, stereotypes, myoclonus, dystonia, tremor, etc. (Blackburn and Parnes, 2021). Idiopathic tremor is the most common movement disorder, with a prevalence rate of 14 and 21% in older adults over 65 years of age and in children and adolescents (Moghal et al., 1994; Kurlan et al., 2002), respectively. However, movement disorders are not only a major area of Neurology but also play an equally large role in geriatrics and related fields (Haunton, 2019). Parkinson’s disease is a neurological disorder and according to the statistics of The Global Burden of Disease Study, there is currently a considerable increase in the number of patients manifesting Parkinson’s disease, which is projected to reach more than 14.2 million by 2040 (Pringsheim et al., 2014; Dorsey and Bloem, 2018).

Clinical manifestations of movement disorders are complicated. Different types of movement disorders require the application of different pharmacological treatments, for example, in patients with dystonia who also have symptoms of mild ataxia, greater attention should be paid to hereditary causes, and in patients with chorea whose symptom cause is unknown, genetic testing for Huntington disease should be performed after appropriate consultation for the initial diagnosis and then other diagnostic and therapeutic workups (Abdo et al., 2010). Pharmacological treatment is currently the main focus on movement disorders in neurological disorders, such as Parkinson’s disease, and to some extent relieves its symptoms using pharmacological treatment. However, movement-based movement disorders often do not respond well to pharmacological treatments (Poewe et al., 2017) and the therapeutic window narrows as treatment progresses. Exercise helps improve the brain’s ability to repair itself and neuroplasticity, influence motor function recovery after brain injury, and prevent various neurodegenerative diseases (Smith and Zigmond, 2003; van der Kolk et al., 2018).

Bibliometric analysis used econometric research methods to study a body of literature and quantify the past published pieces of literature (Döbrössy and Dunnett, 2003). In recent years, there has been a gradual increase in research on non-pharmacological-based exercise therapy, however, no studies for bibliometric analysis were conducted in this field. Therefore, this study is a systematic bibliometric analysis of the field. The CiteSpace software was used to analyze the current state of research on exercise-based non-pharmacological treatments for movement disorders between 2010 and 2021, including annual publication volume, countries, institutions, journals, authors, keywords, and research directions, to provide future researchers guide of hot spots and directions. The New Horizons expressed in their article an encouragement of the development of new research and therapies and is of great value to the therapeutic and the multidisciplinary cross-learning fields (Wang et al., 2020).

## Materials and methods

**FIGURE 1:**
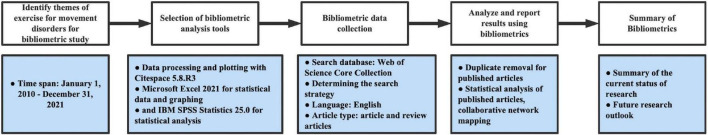
Research flow chart.

The flow chart of this research is shown in Figure 1.

### Search strategy

All literature was obtained from the Web of Science (WOS). The current study showed that the WOS database provides more detailed data in analyzing published articles (Yeung, 2019), and it is more accurate than the Scopus database in terms of criteria based on cited literature, journal classification (Wang and Waltman, 2016), etc. The search formula was determined using Medical Subject Headings (MeSH), which provides a more comprehensive biomedical vocabulary and is a good resource for interpreting biomedical data (Yu, 2018). After determining the search formula, Topic, Title, and Abstract searches were conducted on the WOS (Donthu et al., 2021), and Title searches were selected to ensure the validity of the data source because of the large number of non-relevant literature under Topic and Abstract searches. The search strategy was set to all titles, as follows: (TI = [movement disorder OR dyskinesia syndrome OR myopathies OR myotonic OR myopathy OR paramyotonia congenita OR eulenburg disease OR tremor OR quiver OR rigidity OR rigidities OR gegenhalten OR hypodynamia OR bradykinesia OR myoclonus OR myoclonic OR ataxia OR ataxy OR coordination impairment OR dyssynergia]) AND TI = (exercise OR activity OR activities OR physical OR activity OR water sports OR motor control OR isometric OR aerobic OR train OR sport OR strength OR athletic OR movement OR endurance OR walk OR yoga OR stretch OR kinesiotherapy OR resistance OR pilates OR hydrotherapy OR stability OR tai chi OR core control OR swim OR Sprint OR martial art OR dance OR run). English articles accounted for 96% of all articles retrieved, and both Average per item (20.58) and h-index (92) for English articles were substantially higher than Average per item (2.84) and h-index (8) for non-English articles. English articles were selected to ensure the quality of the included data sources. The language of the articles in the search was set to English, the type of articles was set to “articles and review articles,” and the time was set to from January 1, 2010 to December 31, 2021. Data were cleaned to ensure the quality of the included literature, as WOS is not a database dedicated to bibliometric analysis (Donthu et al., 2021). CiteSpace was used to de-duplicate all retrieved literature and remove invalid literature, resulting in the inclusion of a total of 2,626 papers. In bibliometric analysis, the amount of data incorporated should be sufficiently large, e.g., greater than 500 articles, otherwise the use of bibliometric analysis is not required (Donthu et al., 2021). The final search and download date was May 20, 2022.

### Analysis tools

The CiteSpace 5.8.R3, Microsoft Excel 2021, and IBM SPSS Statistics 25.0 software were used for plotting and statistical analysis. The CiteSpace 5.8.R3 software is based on the Java platform. The Java platform is a flexible tool for data analysis and it allows data visualization. The software has a unique ability in analyzing the generation of the country, institution, author, keyword, clustering analysis, etc. The visualization mapping function can detect the trends and hotspots of visualization literature through systematic search. It analyzed the association and cooperation networks among countries, institutions, authors, explore the trends of keywords, and clustering analysis, subsequently presenting them through visualization mapping (Synnestvedt et al., 2005). CiteSpace can be analyzed and made into 8 different visualizations. The researcher can set different time periods, nodes, and thresholds in the interface and select different nodes for analysis, nodes such as countries, institutions, journals, authors, etc. (Fang et al., 2018). The visual graph mainly consists of nodes and links and represents different elements such as countries, institutions, etc. Link acts as the main bridge among nodes and interconnects them to form a network relationship. The shades of node and link colors represent the number of references, with darker colors representing the increased number of references. Centrality represents the degree of connection among unrelated nodes, therefore, nodes with higher centrality are recognized as important nodes or key turning points in a domain (Pei et al., 2019), meanwhile, nodes with centrality values greater than 0.1 are considered as key nodes. The CiteSpace software also allows geospatial visualization mapping of the literature (Chen, 2017). The analysis of co-cited journals, authors, references, and keywords can provide clues to the development of the field of expertise (Fang et al., 2018), and is more rigorous and convincing in terms of the development and analysis of trends in the field of study than the analysis of citations alone (Mustafee et al., 2014).

The Microsoft Excel 2021 was used to count and make tables and pictures of literature data, make line graphs of annual publications of literature, and make statistical analyses of countries, institutions, and authors.

Pearson’s correlation analysis between the year of publication and the number of published articles was performed using the IBM SPSS Statistics 25.0 software.

### Data extraction

The bibliometric indicators of published articles were extracted using the search strategy of the WOS Core Collection database. These include Science Citation Index Expanded (SCI-EXPANDED), Social Sciences Citation Index (SSCI). The number of publications, co-citations, open access, and h-index of countries, institutions, and research directions was extracted using the CiteSpace with Microsoft Excel software, the keyword trends were analyzed, and relevant visualization plots were produced.

This study explains the current status and outlook of research on exercise-based non-pharmacological treatments for movement disorders through the following sections.

• Analysis of the distribution and trends of countries, institutions, journals, authors, and disciplines.
• Assess the collaboration network among countries, institutions, and authors.
• Citation, open access, and h-index analysis. Citation frequency analysis reflects the quality of publications. The higher the citation frequency, the better the quality. The h-index evaluates the academic level and assesses the number and output levels of researchers’ academic outputs. The higher the h-index, the greater the influence (Hirsch, 2005). Open access reflects the chance of the article being cited. The higher the open access, the more beneficial it is to promote the dissemination and communication of research results.
• The analysis of keyword highlighting, clustering, and citation literature.

## Results

### Annual publication volume and growth trend analysis

**FIGURE 2:**
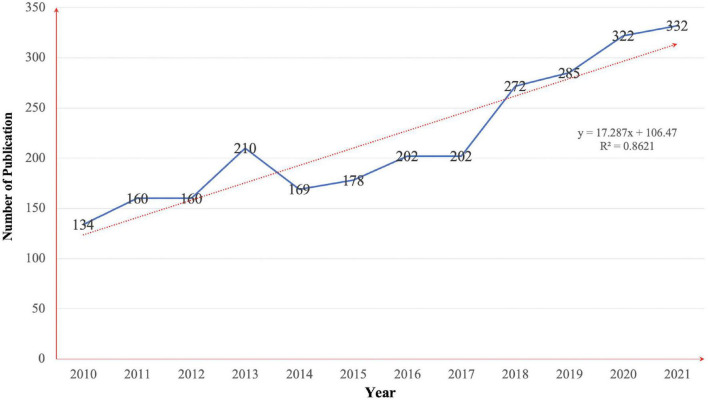
Annual publication volume of published articles.

The annual publication volume is shown in Figure 2. A total of 2,626 articles were included, with a fluctuating growth trend from 2010 to 2017, a gradual growth trend from 2017 to 2021, and a peak in the number of articles published in 2021 (332). The trend of publication volume from 2010 to 2021 shows that exercise-based non-pharmacological treatment for movement disorders is gradually increasing. The American College of Sports Medicine launched the Exercise is Medicine program as early as 2007. It emphasizes on encouraging physicians to promote the treatment and prevention of chronic diseases through scientific exercise when treating their patients, and this program is globally available. The promotion of exercise as a treatment of chronic diseases had a tremendous effect globally (Sallis, 2009), contributing to the rapid growth in publication after 2010. According to a 2015 article, maintaining physical activity is necessary to prevent non-communicable diseases and should increase the role and status of exercise in the medical field (Eijsvogels and Thompson, 2015). The highest number of publications in a single year was reached in 2021, and the development trend in recent years indicates that it is currently in a period of rapid development, in which the movement form is expected to become an important modality in the treatment of movement disorders in the coming years, becoming a mainstream alternative to pharmacological treatment modalities and receiving focused attention from research teams. According to the correlation analysis, the volume of publication was positively correlated with the year (*r* = 0.928, *p* < 0.001).

### Analysis of review articles and articles

The analysis of the types of literature only included theses and reviews. Of the 2,626 articles, 2079 articles (79.17%) and 547 reviews (20.83%) were cited. “Delayed emergence of a parkinsonian disorder or dementia in 81% of older men initially diagnosed with idiopathic rapid eye movement sleep behavior disorder: a 16-year update on a previously reported series (Schenck et al., 2013)” was published in the Journal of *Sleep Medicine* and was one of the top five most cited articles (455) of all articles. As shown in the text, 38% of patients with idiopathic rapid eye movement (REM) sleep behavior disorder are transformed into a Parkinson’s disease after several years. Studies recommended that patients with Parkinson’s disease undergo an annual neurological examination focusing on their cognitive and motor function, and these research advances have significant potential clinical value in the field of multidisciplinary integration (Schenck et al., 2013). The main aspect of diagnosing and treating REM sleep behavior disorder is still medication (Schenck and Mahowald, 2012), and resistance training and self-weight interval training can improve sleep disorders in patients with Parkinson’s disease (Amara et al., 2020). The top three among the review articles (373) in terms of frequency of citations were “The Movement Disorder Society Evidence-Based Medicine Review Update: Treatments for the Motor Symptoms of Parkinson’s Disease (Fox et al., 2011),” Published in *Movement Disorders*. The article’s research illustrates that the training format can reduce the risk of falls, balance, gait, and other abilities in people with Parkinson’s disease. It has a positive effect on the rehabilitation process in moderate-to-high intensity training in patients with Parkinson’s disease. According to past clinical studies, this will be a research trend in the future proving exercise therapy as a treatment for the disease (Fox et al., 2011).

### Analysis of countries and institutions

**FIGURE 3:**
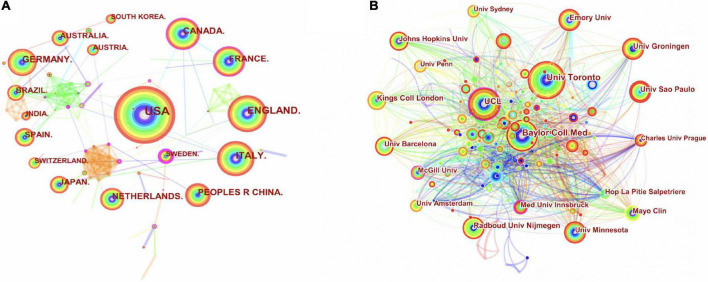
Map of collaborative relationships between countries **(A)** and institutions **(B)** that have published articles. PEOPLES R CHINA, People’s Republic of China; Univ, university.

**FIGURE 4:**
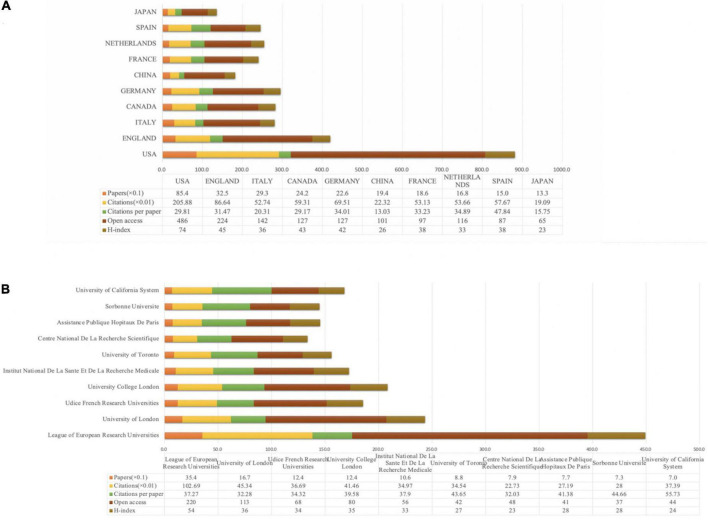
Exercise on the number of publications, citations, average per item, open access, and h-index for countries **(A)** and institutions **(B)** that publish articles in the field of movement disorders.

The CiteSpace and Microsoft Excel software was used to analyze the countries and institutions that published articles in the field, generating plots of countries (Figures 3A, 4A), and institutions (Figures 3B, 4B). In total, 96 countries and 3,058 institutions have published articles in this field in 2,626 articles. According to the number of published articles, the top three countries and regions were the United States (854), the England (325) and Italy (293). According to CiteSpace statistical centrality, the three highest values are in the United States (0.14), Argentina (0.12), and Canada (0.07). In addition to Spain being the highest in average per item (47.84), the United States maintains its lead in the analysis of citations (20,588), open access (486), and h-index (74), based on the combination of publication volume, centrality, citations, average per item, open access, and h-index combined. It showed that the United States maintained its dominant and most influential position in exercise-based non-pharmacological research on movement disorders. The ability of the United States to become a central country in this field of research is closely related to the rapid development of the exercise related non-pharmacological treatment field in the United States, which started early and has received more attention from researchers and has a strong academic research background. Moreover, the United States, Argentina, Canada, Spain, England, and Germany have a strong collaboration with other countries and regions. In the institutional analysis of the published literature, the top three institutions by publication volume are the League of European Research Universities (354), University of London (167), and Udice French Research Universities (124). The top three institutions with the highest centrality values are the University College London (0.15), McGill University (0.12), and Pitie Salpetriere (0.11). The University of California System (55.73) ranked highest in average per item statistics, meanwhile, the League of European Research Universities ranked highest and stayed ahead in publications (354), citations (10,269), open access (220), and h-index (54). Based on the combined analysis of publication volume, centrality, citations, average per item, open access, and h-index, the League of European Research Universities, University College London, and the University of London are the major research institutions. Although the United States is the central country in this field of research, University College London and the University of London, which are major research institutions, are located in the United Kingdom, indicating that in addition to the United States, England in this field of research also maintains a high international influence. Meanwhile, the University of London is second only to the League of European Research Universities in the statistics in terms of the number of publications (167), citations (4,534), open access (113), and h-index (36). The University College London, McGill University, Pitie Salpetriere, University of Toronto, and Baylor College of Medicine have extensive collaborations with other institutions.

### Analysis of journals and co-cited journals

A total of 886 academic journals have been published among the 2,626 articles. A table was created for the top ten journals in terms of the number of articles published (Table 1). The top three journals in terms of the number of articles published were *Movement Disorders* (152), *Parkinsonism & Related Disorders* (85), and *Sleep Medicine* (67). In addition to the *Frontiers in Neurology* journal, which had the highest number of publications in the open access statistics (52).

**TABLE 1:** The top 10 journals in the field of exercise for movement disorders.

Journal	Articles	Times cited (WoS)	Average per item	Open access	WoS sort	IF (2021)	Quartile	H-index
*Movement Disorders*	152	9,435	62.07	49	Clinical Neurology	9.698	Q1	41
*Parkinsonism* & *Related Disorders*	85	1,463	17.21	27	Clinical Neurology	4.402	Q2	22
*Sleep Medicine*	67	1,989	29.69	12	Clinical Neurology	4.842	Q2	23
*Frontiers In Neurology*	52	447	8.6	52	Clinical Neurology; Neurosciences	4.086	Q2	13
*Movement Disorders Clinical Practice*	40	266	6.65	32	Clinical Neurology	4.514	Q2	8
*Sleep*	36	843	23.42	14	Clinical Neurology; Neurosciences	6.313	Q1	32
*Plos One*	33	581	17.61	33	Multidisciplinary Sciences	3.752	Q2	14
*Journal Of Neurology*	32	804	25.13	6	Clinical Neurology	6.682	Q1	18
*Current Neurology And Neuroscience Reports*	27	371	13.74	9	Clinical Neurology; Neurosciences	6.03	Q1	12
*European Journal Of Neurology*	26	342	13.15	8	Clinical Neurology; Neurosciences	6.288	Q1	10

WoS, Web of Science; IF, impact factor.

*Movement Disorders* journals maintained their lead and dominated in terms of publication volume (152), citation frequency (9,435), per-page citation frequency (62.07), impact factor (9.698), and h-index (41). Of the top ten journals, five were located in the first quarter (Q1) of the WOS database classification, and the other five journals are all in the second quarter (Q2), with an average impact factor of 5.6607. The quarterly and impact factors show that all included articles are highly referenced.

**FIGURE 5:**
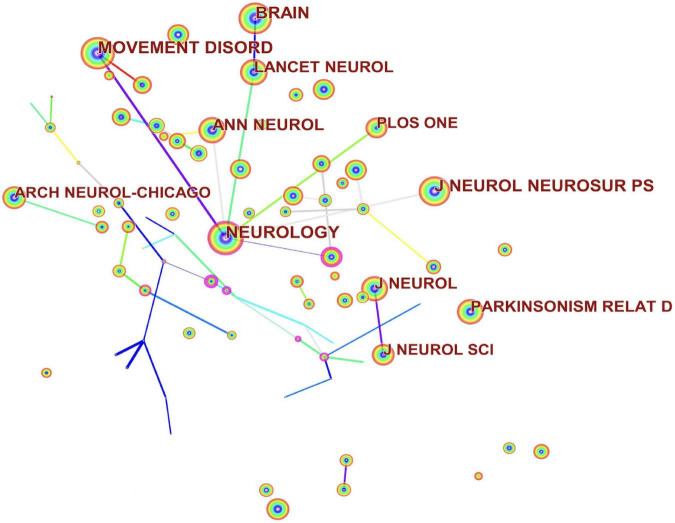
Journal co-citation maps in the field of exercise for movement disorders research.

Co-cited journals were made into a graph by CiteSpace software (Figure 5). The top three journals in terms of the number of co-citations were *Neurology* (1,455), *Movement Disorders* (1,408), and *Brain* (1,121). The top three journals according to centrality were *Archives of Physical Medicine and Rehabilitation* (0.04), *Psychopharmacology* (0.04), and *Journal of Pharmacology and Experimental Therapeutics* (0.04). *Movement Disorders* journals are the leading journals in the field of exercise-based non-pharmacological research based on publication volume, co-citations, and centrality on movement disorders and provide important contributions to research in the field. The top three journals ranked according to their centrality and have values of less than 0.1 are not considered journals with a key role.

### Authors and co-cited authors

**FIGURE 6:**
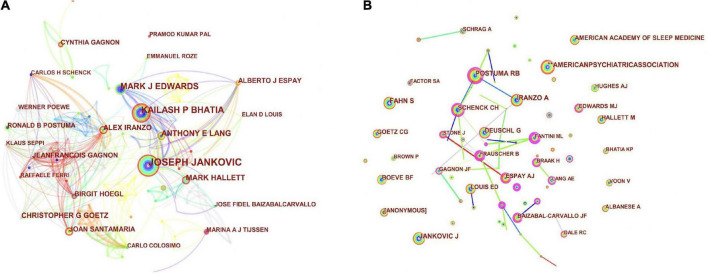
Collaborative network map of authors **(A)** and co-cited authors **(B)** of exercise for movement disorders.

To fully explore the influential authors in the field and show the collaborative networks among authors, the authors and co-cited authors of the published literature were made into a map using the CiteSpace software (Figure 6). A total of 486 nodes and 1,206 connections appeared in the analysis of authors (Figure 6A), meanwhile, 744 nodes and 4,599 connections appeared in the analysis of co-cited authors (Figure 6B). The larger nodes represent greater roles in the overall network mapping. Authors of published articles are summarized and co-citation frequency and centrality are displayed (Supplementary Table 1). The top ten authors, co-cited authors, and co-cited literature were indicated in Table 2. The top three authors with the most published articles were Jankovic, Joseph (45), Bhatia, Kailash P. (40), and Hoegl, Birgit (32). Baylor College of Medicine’s Professor Jankovic, Joseph has the highest number of publications and his research interests are focused on Neurosciences Neurology, particularly on neurological disorders of the elderly. Among his cited articles, the top five of which are four on the Parkinson’s disease (Hong et al., 2010; Marks et al., 2010; Marek et al., 2011; Nalls et al., 2019) and one on the movement disorders (Albanese et al., 2013). A close collaboration has been established among authors such as Fidel Baizabal-Carvallo, Jose, Hallett, Mark, Mehanna, and Raja. According to the CiteSpace analysis, the top three authors with the highest centrality ranking were Bhatia, Kailash P. (0.08), Postuma, Ronald B. (0.05), and Dauvilliers, Yves (0.05), all with centrality values of less than 0.1, which is insufficient as a critical node, indicating that the intensity of collaboration among researchers was not high.

**TABLE 2:** The top 10 authors, co-cited authors, and co-cited references on exercise for movement disorders.

Author	Published articles	Co-cited author	Cited times	Co-cited reference	Cited times
Jankovic, Joseph	45	Postuma, Ronald B.	257	Idiopathic REM sleep behavior disorder and neurodegeneration—an update	41
Bhatia, Kailash P.	40	Iranzo, Alex	243	Delayed emergence of a parkinsonian disorder or dementia in 81% of older men initially diagnosed with idiopathic rapid eye movement sleep behavior disorder: a 16-year update on a previously reported series	40
Hoegl, Birgit	32	Fahn, Stanley	233	Neurodegenerative disease status and post-mortem pathology in idiopathic rapid-eye-movement sleep behavior disorder: an observational cohort study	39
Edwards, Mark J.	31	American Psychiatric Association	232	Quantifying the risk of neurodegenerative disease in idiopathic REM sleep behavior disorder	35
Iranzo, Alex	31	Jankovic, Joseph	200	MDS clinical diagnostic criteria for Parkinson’s disease	30
Postuma, Ronald B.	30	Schenck, Carlos H.	197	Phenomenology and classification of dystonia: A consensus update	29
Hallett, Mark	28	American Academy of Sleep Medicine	169	Idiopathic rapid eye movement sleep behavior disorder: diagnosis, management, and the need for neuroprotective interventions	29
Santamaria, Joan	28	Boeve, Bradley F.	167	Neurodegenerative Disorder Risk in Idiopathic REM Sleep Behavior Disorder: Study in 174 Patients	29
Gagnon, Jean-Francois	27	Deuschl, Guenther	150	Parkinson risk in idiopathic REM sleep behavior disorder Preparing for neuroprotective trials	27
Mirelman A	20	[Anonymous]	134	Polysomnographic diagnosis of idiopathic REM sleep behavior disorder	25

The top three authors in terms of co-citations were Postuma, Ronald B. (257), Iranzo, Alex (243), and Fahn, Stanley (233). Professor Postuma, Ronald B. of McGill University has the highest number of co-citations, with a research interest in Neurosciences Neurology. The top three authors according to centrality are Postuma, Ronald B. (0.12), Iranzo, Alex (0.04), and Fahn Prof. Postuma, Ronald B. Professor Postuma, Ronald B. is the key author of the co-citation and has also established close collaboration with authors such as Gagnon, Jean-Francois, and Montplaisir, Jacques Y.

### Analysis of subject categories of Web of Science

**FIGURE 7:**
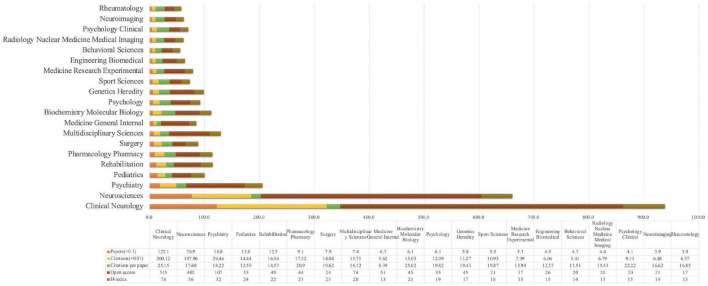
Analysis of the number of publications, citations, average per item, open access, and h-index for exercise for subject categories in the field of movement disorders.

In the analysis of 2,626 articles on the study of movement disorders with exercise-based non-pharmacological treatments, a total of 145 disciplinary categories were classified, and the top 20 disciplinary categories with the highest number of publications were charted (Figure 7). Clinical Neurology (1,221) and Neurosciences (769) accounted for 75.781% of the total number of publications, making them the two leading disciplines in this field of research. Clinical Neurology is leading in the number of publications (1,221), citations (20,012), average per item (25.15), open access (515), and h-index (76). There are 90 research directions in 145 subject categories, mainly in Neurosciences Neurology, Psychiatry, and Psychology. The Neurosciences Neurology research directions accounted for 58.72% of the publication volume (1,542). The disciplinary classification is dominated in the field of neurological related research, but also forms a multidisciplinary network of intersectional relationships, including pediatrics, rehabilitation, and surgery. It provides a background of one-discipline dominated multidisciplinary intersection for research in the field of exercise to movement disorders.

### Analysis of co-cited and cited references

**FIGURE 8:**
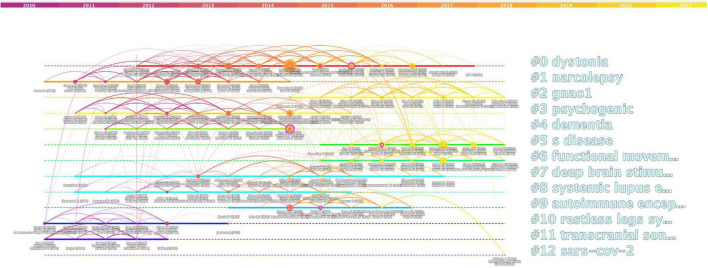
Timeline view map of exercise for movement disorders.

Statistical analysis of co-cited references is an important indicator for correlation analysis in the literature. The higher the number of co-cited references, the higher the correlation among documents. Figure 8 shows a timeline plot of co-cited references, which shows 12 clusters, all of which were extracted by keywords of co-cited references. Dystonia, narcolepsy, and gnao1 were labeled as the first cluster #0, second cluster #1, and third cluster #2, respectively. In the analysis of co-cited literature (Table 2), “Idiopathic REM sleep behavior disorder and neurodegeneration-an update (Högl et al., 2018)” had the highest number of co-citations (41), meanwhile, “The spectrum of movement disorders in children with anti-NMDA receptor encephalitis (Baizabal-Carvallo et al., 2013)” had the highest centrality value (0.15).

The top ten most cited articles are shown in Table 3. The “Diagnostic criteria for mild cognitive impairment in Parkinson’s disease: Movement Disorder Society Task Force guidelines (Litvan et al., 2012)” had the highest number of citations (1,365), however, all citations were in the Clinical Neurology disciplinary classification, meanwhile, in the Neurosciences as the second most cited disciplinary classification, “The Treatment of Restless Legs Syndrome and Periodic Limb Movement Disorder in Adults-An Update for 2012: Practice Parameters with an Evidence-Based Systematic Review and Meta-Analyses (Aurora et al., 2012)” holds the highest citation frequency (209). “Clinical Diagnosis of Progressive Supranuclear Palsy: The Movement Disorder Society Criteria (Höglinger et al., 2017)” ranked first in citations in each of the last two years with the highest annual average of citation frequency (127.83).

**TABLE 3:** The top 10 articles with the most citations on exercise for movement disorders.

Title	First Author	Journal	IF (2021)	Year	Citations (WoS)	WoS Sort
Diagnostic criteria for mild cognitive impairment in Parkinson’s disease: Movement Disorder Society Task Force guidelines	Litvan, Irene	*Movement Disorders*	9.698	2012	1365	Clinical Neurology
Multifunctional wearable devices for diagnosis and therapy of movement disorders	Son, Donghee	*Nature Nanotechnology*	40.523	2014	980	Materials Science
Clinical Diagnosis of Progressive Supranuclear Palsy: The Movement Disorder Society Criteria	Hoeglinger, Guenter U.	*Movement Disorders*	9.698	2017	762	Clinical Neurology
The Movement Disorder Society Evidence-Based Medicine Review Update: Treatments for the Non-Motor Symptoms of Parkinson’s Disease	Seppi, Klaus	*Movement Disorders*	9.698	2011	539	Clinical Neurology
How Common Is the Most Common Adult Movement Disorder? Update on the Worldwide Prevalence of Essential Tremor	Louis, Elan D.	*Movement Disorders*	9.698	2010	514	Clinical Neurology
Consensus Statement on the Classification of Tremors. From the Task Force on Tremor of the International Parkinson and Movement Disorder Society	Bhatia, Kailash P.	*Movement Disorders*	9.698	2018	474	Clinical Neurology
Delayed emergence of a parkinsonian disorder or dementia in 81% of older men initially diagnosed with idiopathic rapid eye movement sleep behavior disorder: a 16-year update on a previously reported series	Schenck, Carlos H.	*Sleep Medicine*	4.842	2013	455	Neurosciences Neurology
Neurodegenerative disease status and post-mortem pathology in idiopathic rapid-eye-movement sleep behavior disorder: an observational cohort study	Iranzo, Alex	*Lancet Neurology*	59.935	2013	400	Neurosciences Neurology
How to identify tremor dominant and postural instability/gait difficulty groups with the movement disorder society unified Parkinson’s disease rating scale: Comparison with the unified Parkinson’s disease rating scale	Stebbins, Glenn T.	*Movement Disorders*	9.698	2013	379	Clinical Neurology
The Movement Disorder Society Evidence-Based Medicine Review Update: Treatments for the Motor Symptoms of Parkinson’s Disease	Fox, Susan H.	*Movement Disorders*	9.698	2011	373	Clinical Neurology

### Analysis of co-citation keywords and emergent keywords

**FIGURE 9:**
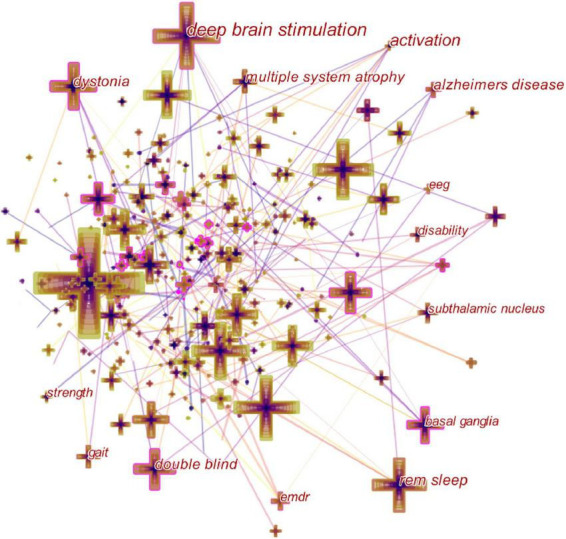
Keyword map of exercise for movement disorders.

The intensity values of co-cited keywords, emergent keywords, and the intensive time of emergent keywords reflect the research frontiers and development trends and uncover emerging research frontiers and development directions. The co-cited keywords were analyzed using the CiteSpace software and created a keyword network map (Figure 9), which generated 488 nodes and 3,996 connections. All keywords were summarized and co-citation frequency and centrality were shown (Supplementary Table 2). The top five keywords in terms of co-citation frequency were Parkinson’s disease (557), movement disorder (355), deep brain stimulation (166), diagnosis (156), and REM sleep (145). The top five keywords with centrality values were brain (0.07), performance (0.06), clinical feature (0.06), movement disorder (0.05), and restless legs syndrome (0.05), all of which had centrality values of less than 0.1 and were not used as important references. Parkinson’s disease, movement disorder, deep brain stimulation, diagnosis, and REM sleep were hot keywords in terms of co-citation frequency and centrality analysis, indicates widespread interest in the study of movement therapy for movement disorders.

**FIGURE 10:**
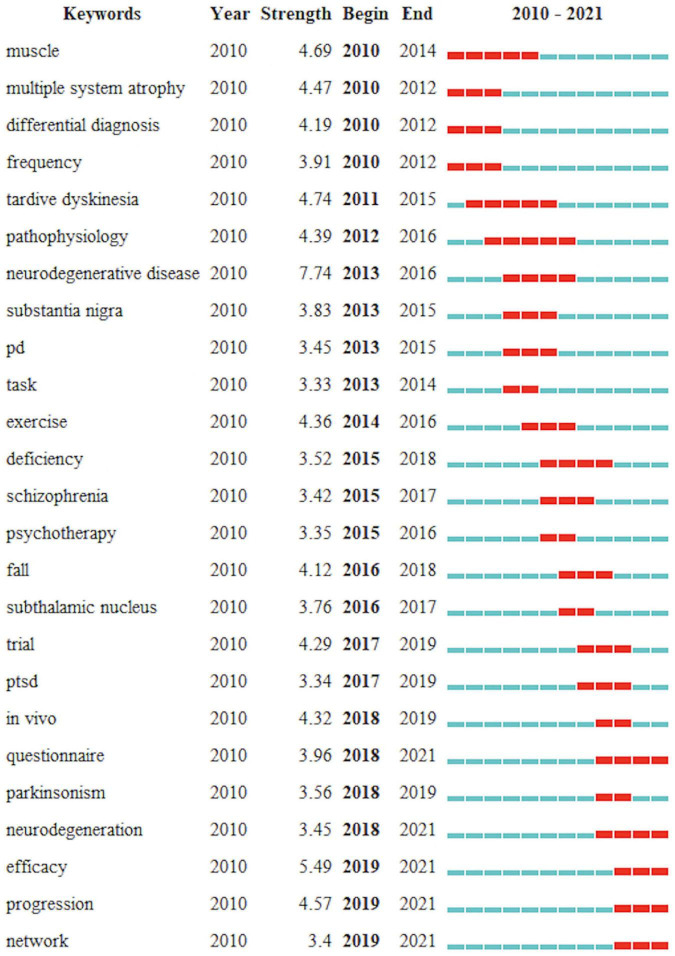
The top 25 keywords with the strongest burst value. Red bar represents keywords with higher co-citation in the time period.

For a more in-depth analysis of the keywords, the burst value and burst time of keywords were analyzed using the CiteSpace software (Figure 10). The burst value of a keyword represents that it has received special attention at a certain time and represents the research hotspot in that research area at a certain time (Lin et al., 2020). Figure 10 shows the 25 keywords with the highest intensity of burst values, with the red nodes representing the aggregation of time. The three keywords with the highest burst value intensity from 2010 to 2016 are neurodegenerative diseases (7.74), delayed movement disorders (4.74), and muscle disorders (4.69). The top three keywords with the highest burst values from 2017 to 2021 shifted to efficacy (5.49), progression (4.57), and *in vivo* (4.32), however, these three keywords are mainly focused on 2019, keywords indicating that the frontiers in the last five years were focused on therapeutic approaches, research progress, and clinical treatment.

## Discussion

### Global research trends and frontiers in exercise for movement disorders

The present analysis was performed on articles studying exercise therapy for movement disorders published between 2010 and 2021. A total of 2,626 articles were retrieved and these data were entered into the CiteSpace software with various bibliometric indicators extracted for countries, institutions, journals, authors, references, and keywords. Using the CiteSpace and Microsoft Excel software, the data were analyzed and processed and their corresponding graphs and plots were produced.

In the analysis of publication volume, the results show that there is an overall upward trend with increasing years, with a total of 52,888 citations from 2012 to 2021, with an average citation frequency of 20.14 per article, open access 1290, and h-index 91. In 2021, the highest publication volume and citation frequency were 332 and 10,032, respectively. The publication volume rapidly increased from 2016 to 2021, with an increase of 159.7% compared to the volume from 2010 to 2015. However, a sudden increase in the number of publications over a certain time does not guarantee an increase in the quality of research in the field. It is because the data extracted from published articles only counts the number of publications of research in the field and does not analyze the quality of research articles. The frequency of citations of the published articles only represents the size of the attention that researchers give to the articles. In the analysis of the countries and institutions that have published articles, there are 96 and 3,058 that have published articles in this field, respectively. The United States is the leading influential country in this field, with the League of European Research Universities, University College London, and University of London as the main research institutions. In the analysis of journals that published articles, 886 academic journals have published articles in the field, with the *Movement Disorders* journals dominating all journals that published articles. The number of publications of the *Movement Disorders* journals rapidly increased from 2017 to 2020, reaching the highest single-year publication volume in 2019 and 2020 (18) and the highest single-year citation frequency in 2021 (1,898), indicating that the *Movement Disorders* journals have received certain attention and influence in the field in recent years. In previous studies, great progress has been made in diagnosing movement disorders (Sharma, 2020). A growing number of studies have demonstrated that improvement in movement disorders positively affects the patients’ quality of life, however, surgical treatment can have certain side effects, such as residual motor symptoms after surgery (Rowland et al., 2017). Parkinson’s disease is mostly treated with pharmacological and surgical interventions, although they can improve and relieve symptoms, they also produce certain side effects (Ventura et al., 2015). In addition, avoiding surgery in patients and trying to uncover better strategies for the treatment of movement disorders is today’s important long-term goal (Rowland et al., 2017). In the analysis of the authors of the published articles, Prof. Jankovic, Joseph had the highest number of publications (45) and Prof. Postuma, Ronald B. had the highest number of co-citations (257). Both authors have the same research interest in Neurosciences Neurology.

In an analysis of the WOS disciplinary classification, the most heavily concentrated research on exercise for movement disorders is in the Neurosciences Neurology research area of the Clinical Neurology discipline, and this is dominated by the United States and England. The American Medical Association and the American College of Sports Medicine jointly organized the “Exercise is Medicine” advocacy campaign in 2007 to strongly advocate the importance of exercise for health. It was implemented nationwide in the United States, encouraging people of all ages, genders, and ethnicities to maintain good exercise habits. The project established the importance of exercise in health care (Lobelo et al., 2014). The British Journal of Sports Medicine, ranked number one in sports science and has a major influence in the field of sports medicine. The 2,014 article showed that the Exercise is Medicine campaign has now partnered with 39 countries worldwide and in the future exercise therapy will be integrated into the global development strategy of healthcare, making a significant contribution to the global sports medicine field (Lobelo et al., 2014). Exercise therapy is cost-effective and can be widely practiced at the societal level. Studies have demonstrated that exercise improves cognitive performance and outperforms medication as treatment and it has a high positive effect on various neurological disorders. Therefore, researchers and medical practitioners should actively promote exercise therapy to patients with movement disabilities and the need for multidisciplinary collaboration (Nagamatsu et al., 2014).

In an analysis of references, the review of “Idiopathic REM sleep behavior disorder and neurodegeneration-an update” shows the importance of using polysomnography in diagnosing sleep disorders. As explained in other studies (Amara et al., 2020), the improvement of sleep disorders in patients with Parkinson’s disease by exercise can be properly diagnosed by polysomnography. Exercise is potential for the prevention and rehabilitation of various diseases, however, it is currently not fully implemented as treatment in the clinical practice. In the future, exercise should be combined with medicine to be applied as therapy to patients on an equal footing with other medical interventions (Vuori et al., 2013). In recent years, from the keywords that have received great attention, it can be seen that the Parkinson’s disease, movement disorders, deep brain stimulation, efficacy, progression, and *in vivo* have been the hot spots of research on movement disorders by exercise. So far, the main modalities for exercise-based non-pharmacological treatment of movement disorders include tai chi, balance, resistance, and interval training. These modalities have produced different efficacy for different movement disorders through different forms of exercises (Li et al., 2012; Pompeu et al., 2012; Corcos et al., 2013; Amara et al., 2020). The 2018 Physical Activity Guidelines for Americans recommend that people of all ages should maintain varying levels of exercise by maintaining physical activity for health benefits and disease prevention, including special populations with Parkinson’s disease, Alzheimer’s disease, stroke, and cerebral palsy (Piercy et al., 2018). However, taking medications as a treatment for some movement disorders such as tremor, dystonia, and Parkinson’s disease can also be a cause of further movement disorders (Burkhard, 2014; Mehta et al., 2015). It is necessary to explore and investigate the non-pharmacological and non-invasive interventions in the treatment of current movement disorders (Ventura et al., 2015).

### Analysis of hot keywords for Parkinson’s disease and movement disorders

Parkinson’s disease: Parkinson’s disease is mainly suffered by the elderly, and the prevalence increases with age (Pringsheim et al., 2014), the motor symptoms of Parkinson’s disease can be relieved by medication, but inevitably there are certain side effects (van der Kolk et al., 2018). Many studies have now confirmed that the motor symptoms of Parkinson’s can be effectively improved by aerobic exercise, Tai Chi, and resistance training (Li et al., 2012; Pompeu et al., 2012; Corcos et al., 2013; van der Kolk et al., 2018; Marusiak et al., 2019), and even in older adults with other chronic diseases, the risk of chronic disease can be similarly reduced by physical activity (Piercy et al., 2018). In studies of patients with Parkinson’s disease, 74–98% of patients were affected by sleep disorders (Amara et al., 2020), and good sleep is essential for mental and physical health (Buysse, 2014). Sleep disorders can have health consequences if not treated, such as obstructive sleep apnea, insomnia, chronic sleep insufficiency, etc. (Cho and Duffy, 2019). But few medications can effectively improve Parkinson’s sleep disorder, and the existing medication may bring certain side effects, there are studies have proved that resistance training and interval training can improve the sleep disorder of Parkinson’s disease patients, and improve the quality of sleep. In the future, non-pharmacological treatments such as exercise will be an alternative treatment for Parkinson’s sleep disorders (Amara et al., 2020).

Movement disorders: In previous research concepts, dyskinesia was usually considered as an impairment in the ability to control movement triggered mainly by basal ganglia dysfunction, but nowadays, as relevant research continues to develop, the sensory system plays an important role in dyskinesia, especially peripheral sensory feedback. The regulation of proprioception is defective in Parkinson’s disease patients (Patel et al., 2014), and motor deficits caused by gait and balance abilities have a greater impact on Parkinson’s disease patients (Coelho et al., 2010), mostly due to the integration of deficits in multiple feedback systems of visual, auditory, and proprioceptive, leading to motor deficits (Wright et al., 2010; Martens and Almeida, 2012; Sacrey and Whishaw, 2012). Motor training with response stimuli, movement techniques, and other targeted to the relevant systems can overcome movement disorders such as gait (Van Gerpen et al., 2012). Studies in patients with Akathisia and restless legs syndrome have also shown that symptoms can be effectively relieved by continuous exercise. In future studies, research on the effects of the integrated effects of multiple feedback systems on movement disorders should be strengthened (Patel et al., 2014). There is evidence that a simple 12-week low to moderate intensity walking training program in patients with functional movement disorders significantly improves symptoms of movement disorders (Dallocchio et al., 2010). A growing body of research demonstrates that the nervous system can be protected, adapted, regenerated, and neurologically improved through training programs (Ang and Gomez-Pinilla, 2007), suggesting that cognitive-behavioral therapy combining physical movement and cognitive interventions can provide an easy, effective, and feasible means of treating symptoms (Sharpe et al., 2011). In intervention studies of exercise on cognitive performance, attention, and social behavior in movement disorders, regular participation in regular exercise in children with attention deficit hyperactivity disorder was effective in improving cognitive function, attention, and social behavior, and in improving motor performance, thereby reducing motor symptoms (Medina et al., 2010; Den Heijer et al., 2017; Christiansen et al., 2019) and there is also evidence that multidisciplinary crossover approaches such as physical combined with psychotherapy can be positive for some patients (Schrag et al., 2004), and compared to pharmacotherapy is more economical, efficient and practicable compared to medication. In a controlled trial, just 5 days of intensive physical therapy improved more than 60% of patients (Czarnecki et al., 2012). And many cognitive-behavioral treatment programs can be easily combined with exercise forms (Otto et al., 2007). Patients with movement disorders should be targeted in their training programs in the same way as medication programs, which may produce very different symptoms and have a different impact on life even when suffering from the same degree of disorder (Czarnecki et al., 2012). In future research, multidisciplinary cooperation on movement disorder-related disorders, such as neurology, geriatrics, psychology and physical therapy such as exercise should be extensively strengthened to conduct collaborative research and explore more scientific and effective treatment options in the field of movement disorders.

### Strengths and limitations

This study is the first bibliometric analysis based on the WOS Core Collection database summarizing the trends and frontiers of exercise-based non-pharmacological treatments for movement disorders research in the world from 2010 to 2021. A total of 2,626 articles were counted from 96 countries and 886 academic journals. The data of the analyzed articles were more comprehensive, counting the number of publications, countries, institutions, journals, authors, literature types, references, research directions, and keywords.

This study also has limitations in the search strategy because it was only for the WOS Core Collection database, non-English published articles were not included, and only referred to articles and reviews which may have biased the analysis results based on these factors. The results of articles with higher citation frequencies are already known, meanwhile, articles with newer publication years, high influence, and high quality may not have higher citation frequencies.

## Conclusion

The CiteSpace software was mainly used in this study to show the trends in the field of exercise based non-pharmacological treatment for movement disorders research from 2010 to 2021. It also shows the collaborative network among countries, institutions, journals, and authors, and the in-depth analysis of article types, development directions, references, and hot keywords. The present bibliometric analysis provides a new research perspective on the research progress in this field. The research on exercise for movement disorders is receiving a lot of attention, and many research teams are conducting high quality randomized controlled trials to promote the rapid development of the field. Although there are some limitations of the study, it provides a more comprehensive analysis of the research trends and frontier hotspots in the field of non-pharmacological treatment of movement disorders research. The results of the analysis are conducive to more and future promising research in the field of movement therapy for movement disorders.

## Data availability statement

The original contributions presented in this study are included in the article/Supplementary material, further inquiries can be directed to the corresponding authors.

## Author contributions

J-WC wrote the article and data analysis. YG, Y-LZ, and KZ contributed to the conception of the study and helped further revise the article. All authors contributed to the article and approved the submitted version.
