# Supplementary material for: Research trends and frontiers in exercise for movement disorders: A bibliometric analysis of global research from 2010 to 2021
Source: Front Aging Neurosci. 2022 Sep 7;14:977100. doi: 10.3389/fnagi.2022.977100 (PMC9491729; doi:10.3389/fnagi.2022.977100)
Supplement: Supplementary file 1 [file Data_Sheet_1.docx]

**Supplementary File 1** Search strategy for Web of Science

#1 TI=(exercise OR activity OR activities OR physical OR activity OR water sports OR motor control OR isometric OR aerobic OR train OR sport OR strength OR athletic OR movement OR endurance OR walk OR yoga OR stretch OR kinesiotherapy OR resistance OR pilates OR hydrotherapy OR stability OR tai chi OR core control OR swim OR Sprint OR martial art OR dance OR run)

#2 TI=(movement disorder OR dyskinesia syndrome OR myopathies OR myotonic OR myopathy OR paramyotonia congenita OR eulenburg disease OR tremor OR quiver OR rigidity OR rigidities OR gegenhalten OR hypodynamia OR bradykinesia OR myoclonus OR myoclonic OR ataxia OR ataxy OR coordination impairment OR dyssynergia)

#3 #1 AND #2

Timespan=2010.01.01-2021.12.31 Databases=Web of Science Core Collection.

LANGUAGE: (English)

Document type: article and review
