# Supplementary material for: Research trends and frontiers in exercise for movement disorders: A bibliometric analysis of global research from 2010 to 2021
Source: Front Aging Neurosci. 2022 Sep 7;14:977100. doi: 10.3389/fnagi.2022.977100 (PMC9491729; doi:10.3389/fnagi.2022.977100)
Supplement: Supplementary file 2 [file Table_1.docx]

| **Supplementary Table 1** Summary of co-citation frequency and centrality of exercise for movement disorder-related authors. | | | |
| --- | --- | --- | --- |
| **Co-citation frequency** | **Centrality** | **Year** | **Author** |
| 257 | 0.12 | 2010 | Postuma RB |
| 243 | 0.04 | 2010 | IRANZO A |
| 233 | 0.02 | 2010 | Fahn S |
| 232 | 0.04 | 2010 | American PsychiatricAssociation |
| 200 | 0.03 | 2010 | Jankovic J |
| 197 | 0.02 | 2010 | SCHENCK CH |
| 169 | 0.04 | 2010 | American Academy of Sleep Medicine |
| 167 | 0.01 | 2010 | Boeve BF |
| 150 | 0.03 | 2010 | Deuschl G |
| 134 | 0 | 2010 | [Anonymous] |
| 123 | 0.02 | 2010 | Goetz CG |
| 119 | 0.03 | 2010 | Louis ED |
| 119 | 0.08 | 2010 | HALLETT M |
| 118 | 0.02 | 2010 | Espay AJ |
| 114 | 0.02 | 2010 | HUGHES AJ |
| 107 | 0.03 | 2010 | Gagnon JF |
| 102 | 0.02 | 2012 | Edwards MJ |
| 90 | 0.09 | 2010 | Schrag A |
| 90 | 0.03 | 2010 | Fantini ML |
| 90 | 0.05 | 2010 | Voon V |
| 88 | 0.04 | 2011 | Braak H |
| 87 | 0.08 | 2013 | Baizabal-Carvallo JF |
| 85 | 0.02 | 2010 | Frauscher B |
| 80 | 0.04 | 2010 | FACTOR SA |
| 80 | 0.03 | 2011 | Stone J |
| 73 | 0.08 | 2010 | LANG AE |
| 73 | 0.03 | 2012 | Albanese A |
| 73 | 0.03 | 2010 | Montplaisir J |
| 66 | 0.04 | 2010 | Stiasny-Kolster K |
| 64 | 0.02 | 2011 | Dale RC |
| 63 | 0.01 | 2010 | Ferri R |
| 62 | 0.03 | 2010 | Bhatia KP |
| 59 | 0.05 | 2010 | Brown P |
| 59 | 0.03 | 2010 | McKeith IG |
| 58 | 0.03 | 2010 | Berg D |
| 57 | 0.01 | 2010 | **WorldHealthOrganization |
| 56 | 0.04 | 2010 | Allen RP |
| 55 | 0.01 | 2010 | Berardelli A |
| 55 | 0.04 | 2010 | MARSDEN CD |
| 54 | 0.05 | 2011 | Albin RL |
| 53 | 0.03 | 2010 | ELBLE RJ |
| 53 | 0.03 | 2013 | Sixel-Doring F |
| 52 | 0.03 | 2011 | BECK AT |
| 51 | 0.01 | 2010 | FOLSTEIN MF |
| 49 | 0 | 2018 | Hogl B |
| 49 | 0.01 | 2011 | Arnulf I |
| 48 | 0.03 | 2010 | Ondo WG |
| 48 | 0.01 | 2011 | Fasano A |
| 48 | 0.02 | 2010 | Gilman S |
| 48 | 0.04 | 2010 | Cardoso F |
| 48 | 0.01 | 2015 | Erro R |
| 46 | 0 | 2012 | Cohen J |
| 46 | 0.01 | 2012 | Litvan I |
| 45 | 0.08 | 2010 | Schneider SA |
| 44 | 0.03 | 2010 | BENABID AL |
| 44 | 0.03 | 2011 | Dalmau J |
| 43 | 0.02 | 2011 | Schwingenschuh P |
| 43 | 0.1 | 2010 | Tan EK |
| 43 | 0.03 | 2011 | OBESO JA |
| 42 | 0.02 | 2012 | Nomura T |
| 42 | 0.02 | 2010 | Iber C |
| 41 | 0.08 | 2011 | Aarsland D |
| 41 | 0.01 | 2011 | Chaudhuri KR |
| 40 | 0.01 | 2018 | Balint B |
| 40 | 0 | 2010 | HOEHN MM |
| 40 | 0.01 | 2012 | Parees I |
| 39 | 0.01 | 2010 | Benito-Leon J |
| 39 | 0.02 | 2010 | Walters AS |
| 37 | 0.01 | 2010 | Williams D T |
| 37 | 0.02 | 2010 | Antonini A |
| 37 | 0 | 2013 | Ferini-Strambi L |
| 36 | 0.01 | 2010 | Kim JS |
| 36 | 0.04 | 2011 | BURKE RE |
| 36 | 0 | 2011 | Schmitz-Hubsch T |
| 35 | 0.01 | 2017 | Fereshtehnejad SM |
| 33 | 0 | 2013 | JOHNS MW |
| 33 | 0.01 | 2010 | Wenning GK |
| 32 | 0 | 2016 | Shapiro F |
| 32 | 0.02 | 2013 | Vidailhet M |
| 32 | 0 | 2016 | Mahlknecht P |
| 31 | 0.01 | 2010 | Ohayon MM |
| 31 | 0.02 | 2010 | Pahwa R |
| 31 | 0.01 | 2011 | Caviness JN |
| 30 | 0.01 | 2013 | Mink JW |
| 30 | 0.01 | 2010 | Winkelman JW |
| 30 | 0 | 2013 | Scherfler C |
| 30 | 0 | 2013 | LORD C |
| 29 | 0.02 | 2015 | Jinnah HA |
| 29 | 0.01 | 2014 | Ilg W |
| 29 | 0.01 | 2010 | ZESIEWICZ TA |
| 29 | 0.01 | 2010 | Eisensehr I |
| 29 | 0.02 | 2013 | Okun MS |
| 28 | 0 | 2012 | Timmermann L |
| 28 | 0 | 2016 | McCarter SJ |
| 28 | 0.01 | 2011 | Gupta A |
| 28 | 0.02 | 2013 | Miyamoto T |
| 28 | 0 | 2016 | Sanger TD |
| 28 | 0.01 | 2013 | Manni R |
| 27 | 0 | 2017 | Wechsler D |
| 27 | 0.01 | 2010 | LAPIERRE O |
| 27 | 0 | 2010 | Adler CH |
| 27 | 0 | 2019 | Nielsen G |
| 26 | 0 | 2011 | Unger MM |
| 25 | 0.01 | 2011 | Trenkwalder C |
| 25 | 0.01 | 2014 | Leigh RJ |
| 25 | 0.01 | 2011 | ALEXANDER GE |
| 25 | 0 | 2016 | Berry RB |
| 25 | 0.01 | 2011 | Comella CL |
| 25 | 0.02 | 2010 | KOLLER WC |
| 25 | 0.01 | 2010 | Hinson VK |
| 24 | 0 | 2017 | Chahine LM |
| 24 | 0.02 | 2012 | Helmich RC |
| 24 | 0.01 | 2013 | Stamelou M |
| 23 | 0.01 | 2011 | Dubois B |
| 23 | 0.03 | 2018 | Gaig C |
| 23 | 0.04 | 2011 | Friedman JH |
| 22 | 0.01 | 2012 | Shibasaki H |
| 22 | 0.01 | 2016 | Maurer CW |
| 22 | 0.02 | 2010 | Vetrugno R |
| 22 | 0.01 | 2018 | Carecchio M |
| 22 | 0.01 | 2013 | Weintraub D |
| 22 | 0.01 | 2012 | Bhidayasiri R |
| 22 | 0.01 | 2013 | De Cock VC |
| 22 | 0 | 2016 | Li SX |
| 21 | 0 | 2015 | Kim HJ |
| 21 | 0.04 | 2011 | Mahowald MW |
| 21 | 0 | 2011 | Massicotte-Marquez J |
| 21 | 0.01 | 2018 | Barber TR |
| 21 | 0.07 | 2015 | Dalakas MC |
| 21 | 0 | 2014 | Kranick S |
| 21 | 0.01 | 2010 | DeLong MR |
| 21 | 0.01 | 2015 | Weaver FM |
| 21 | 0.01 | 2015 | Aybek S |
| 21 | 0.02 | 2010 | Brooks DJ |
| 20 | 0 | 2017 | Poewe W |
| 20 | 0 | 2012 | Vendette M |
| 20 | 0 | 2015 | Marras C |
| 20 | 0.01 | 2011 | GIBB WRG |
| 20 | 0.02 | 2015 | Kupsch A |
| 20 | 0 | 2012 | Mayer G |
| 20 | 0.01 | 2015 | Czarnecki K |
| 20 | 0.01 | 2019 | Rahayel S |
| 20 | 0.01 | 2017 | Ganos C |
| 20 | 0.01 | 2013 | Dauvilliers Y |
| 19 | 0 | 2017 | Rolinski M |
| 19 | 0 | 2010 | Krack P |
| 18 | 0.01 | 2016 | Stefani A |
| 18 | 0.01 | 2017 | Synofzik M |
| 18 | 0.01 | 2011 | Picchietti DL |
| 18 | 0 | 2018 | Hoglinger GU |
| 18 | 0 | 2011 | Anderson KE |
| 18 | 0.02 | 2010 | Blomstedt P |
| 18 | 0.05 | 2019 | Ebrahimi-Fakhari D |
| 18 | 0.01 | 2015 | Armangue T |
| 18 | 0.01 | 2018 | Holtbernd F |
| 18 | 0 | 2017 | Martinez-Martin P |
| 17 | 0 | 2012 | Klein C |
| 17 | 0.01 | 2010 | Benamer HTS |
| 17 | 0.01 | 2015 | Thenganatt MA |
| 17 | 0 | 2017 | Schapira AHV |
| 17 | 0.01 | 2013 | de Lau LML |
| 17 | 0.01 | 2018 | Koy A |
| 17 | 0.01 | 2017 | van der Salm SMA |
| 17 | 0 | 2013 | Nasreddine ZS |
| 17 | 0 | 2011 | WICHMANN T |
| 17 | 0.02 | 2011 | Lu J |
| 16 | 0 | 2015 | Mohammad SS |
| 16 | 0 | 2011 | Emre M |
| 16 | 0.02 | 2010 | BERGMAN H |
| 16 | 0 | 2011 | Olanow CW |
| 16 | 0.01 | 2015 | Volkmann J |
| 16 | 0.01 | 2018 | Martino D |
| 16 | 0 | 2011 | SFORZA E |
| 16 | 0 | 2017 | Arnaldi D |
| 16 | 0 | 2018 | Zhang Y |
| 16 | 0 | 2012 | Aurora RN |
| 16 | 0 | 2011 | Graus F |
| 16 | 0.01 | 2011 | Kurlan R |
| 15 | 0 | 2016 | HAMILTON M |
| 15 | 0 | 2020 | Demartini B |
| 15 | 0 | 2016 | Howell MJ |
| 15 | 0 | 2015 | Romenets Silvia Rios |
| 15 | 0.02 | 2011 | Follett KA |
| 15 | 0 | 2018 | Antelmi E |
| 15 | 0 | 2015 | Tolosa E |
| 15 | 0 | 2015 | Titulaer MJ |
| 15 | 0 | 2012 | Krauss JK |
| 15 | 0 | 2011 | Thomas M |
| 14 | 0 | 2018 | SINGER HS |
| 14 | 0 | 2018 | Calabresi P |
| 14 | 0 | 2016 | Chen DH |
| 14 | 0 | 2013 | Olson EJ |
| 14 | 0 | 2012 | Ferman TJ |
| 14 | 0 | 2011 | Feinstein A |
| 14 | 0.01 | 2018 | Wu P |
| 13 | 0 | 2013 | Hanyu H |
| 13 | 0 | 2015 | Lehericy S |
| 13 | 0.01 | 2014 | Tomlinson CL |
| 13 | 0 | 2013 | Kumru H |
| 13 | 0 | 2010 | Bohannon RW |
| 13 | 0.01 | 2020 | Galbiati A |
| 13 | 0 | 2013 | Manconi M |
| 13 | 0.01 | 2019 | Pagano G |
| 13 | 0.01 | 2014 | Dorsey ER |
| 12 | 0.01 | 2013 | Wing YK |
| 12 | 0.01 | 2019 | Armstrong MJ |
| 12 | 0 | 2020 | Eggink H |
| 12 | 0 | 2018 | Friedman J |
| 12 | 0.02 | 2015 | Bruggemann N |
| 12 | 0 | 2017 | Irani SR |
| 12 | 0.01 | 2010 | Kenney C |
| 12 | 0.01 | 2018 | Pandey S |
| 12 | 0 | 2020 | Heinzel S |
| 12 | 0 | 2017 | Elias WJ |
| 12 | 0 | 2012 | Lee JE |
| 12 | 0 | 2011 | Plazzi G |
| 12 | 0 | 2013 | Boot BP |
| 12 | 0 | 2013 | Siderowf A |
| 12 | 0 | 2011 | Rechtschaffen A |
| 12 | 0 | 2010 | Earley CJ |
| 12 | 0 | 2013 | BUYSSE DJ |
| 12 | 0 | 2011 | Bohnen NI |
| 11 | 0.01 | 2020 | Ben-Pazi H |
| 11 | 0.01 | 2012 | Anheim M |
| 11 | 0 | 2010 | Lyons KE |
| 11 | 0 | 2018 | Meneret A |
| 11 | 0 | 2014 | Kleiner-Fisman G |
| 11 | 0 | 2018 | Mehanna R |
| 11 | 0 | 2017 | Pringsheim T |
| 11 | 0 | 2016 | Wang Y |
| 11 | 0 | 2019 | Moher D |
| 11 | 0 | 2020 | Gelauff J |
| 11 | 0 | 2018 | van Egmond ME |
| 11 | 0 | 2013 | Luppi PH |
| 11 | 0 | 2013 | Sasai T |
| 11 | 0 | 2014 | Shaikh AG |
| 11 | 0 | 2016 | Wijemanne S |
| 11 | 0 | 2018 | Lumsden DE |
| 11 | 0 | 2017 | Rizzo G |
| 11 | 0 | 2017 | Sheehan DV |
| 11 | 0.01 | 2013 | Jellinger KA |
| 10 | 0 | 2014 | Kompoliti K |
| 10 | 0.01 | 2020 | Espay Alberto J |
| 10 | 0 | 2016 | Moro E |
| 10 | 0 | 2016 | Kang SH |
| 10 | 0 | 2012 | Hutchinson M |
| 10 | 0.01 | 2015 | Yang H |
| 10 | 0 | 2017 | Marek K |
| 10 | 0 | 2015 | McKeon A |
| 10 | 0 | 2013 | Oudiette D |
| 10 | 0.01 | 2018 | Cardoso Francisco |
| 10 | 0 | 2017 | Mathieu J |
| 10 | 0 | 2013 | Claassen DO |
| 10 | 0 | 2017 | Hammaren E |
| 10 | 0 | 2013 | Morgante F |
| 10 | 0 | 2013 | Boeve B |
| 10 | 0 | 2011 | Chen R |
| 10 | 0.01 | 2011 | Casey DE |
| 10 | 0 | 2011 | Avanzino L |
| 10 | 0.01 | 2013 | Kunz D |
| 10 | 0 | 2020 | Fernandez-Arcos A |
| 9 | 0 | 2021 | Lundberg IE |
| 9 | 0 | 2013 | Compta Y |
| 9 | 0 | 2010 | Walter U |
| 9 | 0.01 | 2012 | Carbon M |
| 9 | 0 | 2015 | Fox SH |
| 9 | 0 | 2013 | Gjerstad MD |
| 9 | 0 | 2015 | Starr PA |
| 9 | 0 | 2019 | Haba-Rubio J |
| 9 | 0 | 2019 | Marchand DG |
| 9 | 0 | 2017 | Lee JH |
| 9 | 0 | 2010 | Nutt JG |
| 9 | 0 | 2013 | Mazza S |
| 9 | 0 | 2010 | Sugden D |
| 9 | 0 | 2012 | Driver-Dunckley E |
| 9 | 0 | 2020 | Kalia LV |
| 9 | 0.01 | 2018 | Hauser RA |
| 9 | 0 | 2010 | Booij J |
| 9 | 0 | 2014 | Bastian AJ |
| 9 | 0 | 2013 | Kotagal V |
| 9 | 0.01 | 2010 | RAJPUT AH |
| 9 | 0 | 2017 | Politis M |
| 9 | 0 | 2010 | Fernandez HH |
| 8 | 0 | 2011 | Raethjen J |
| 8 | 0 | 2016 | Hamani C |
| 8 | 0 | 2021 | Alexanderson H |
| 8 | 0 | 2013 | Lai YY |
| 8 | 0 | 2021 | Mollenhauer B |
| 8 | 0 | 2012 | Quartarone A |
| 8 | 0 | 2021 | LaFaver K |
| 8 | 0 | 2015 | Schuepbach WMM |
| 8 | 0.01 | 2012 | Poston KL |
| 8 | 0 | 2017 | First MB |
| 8 | 0 | 2013 | Sinforiani E |
| 8 | 0 | 2011 | Tarsy D |
| 8 | 0 | 2012 | Silber MH |
| 8 | 0 | 2011 | Ferrara J |
| 8 | 0 | 2010 | Lee MS |
| 8 | 0 | 2016 | Muller MLTM |
| 8 | 0 | 2011 | Rascol O |
| 8 | 0 | 2020 | Schirinzi T |
| 8 | 0 | 2013 | Neychev VK |
| 8 | 0 | 2021 | Gagnon C |
| 8 | 0 | 2012 | Bliwise DL |
| 8 | 0.02 | 2012 | Alarcon F |
| 8 | 0 | 2018 | Strupp M |
| 8 | 0 | 2011 | Correll CU |
| 8 | 0.01 | 2016 | Bisson JI |
| 8 | 0 | 2018 | Pittock SJ |
| 8 | 0 | 2017 | Beudel M |
| 8 | 0 | 2010 | Vaillancourt DE |
| 8 | 0 | 2011 | Cubo E |
| 8 | 0 | 2018 | Doppler K |
| 8 | 0.01 | 2013 | Bonakis A |
| 7 | 0 | 2021 | Munters LA |
| 7 | 0.01 | 2010 | Dogu O |
| 7 | 0 | 2013 | Limousin N |
| 7 | 0 | 2011 | BONNET MH |
| 7 | 0 | 2018 | Carvajal-Gonzalez A |
| 7 | 0 | 2013 | Jouvet M |
| 7 | 0 | 2017 | Garcia-Borreguero D |
| 7 | 0 | 2018 | Vilas D |
| 7 | 0 | 2012 | Chen JJ |
| 7 | 0 | 2018 | Lancaster E |
| 7 | 0 | 2013 | Onofrj M |
| 7 | 0 | 2012 | Baik JS |
| 7 | 0 | 2010 | Levy R |
| 7 | 0.01 | 2011 | Guy W |
| 7 | 0 | 2021 | Ricciardi L |
| 7 | 0 | 2021 | Nahab FB |
| 7 | 0 | 2011 | Freund HJ |
| 7 | 0 | 2013 | Bugalho P |
| 7 | 0 | 2021 | BOHAN A |
| 7 | 0 | 2010 | Godau J |
| 7 | 0 | 2011 | Ahlskog JE |
| 7 | 0 | 2021 | Burk K |
| 7 | 0 | 2011 | Gouider-Khouja N |
| 7 | 0 | 2012 | Allen R |
| 6 | 0 | 2010 | Breakefield XO |
| 6 | 0 | 2011 | Chervin RD |
| 6 | 0 | 2014 | Blahak C |
| 6 | 0 | 2018 | Sabater L |
| 6 | 0 | 2011 | Chastan N |
| 6 | 0 | 2018 | Shulman LM |
| 6 | 0 | 2021 | Tse W |
| 6 | 0 | 2013 | Tippmann-Peikert M |
| 6 | 0 | 2013 | Stockner H |
| 6 | 0 | 2021 | Cavanna AE |
| 6 | 0 | 2010 | Connor JR |
| 6 | 0 | 2013 | Poryazova R |
| 6 | 0 | 2019 | Dang-Vu TT |
| 6 | 0 | 2020 | Li YY |
| 6 | 0 | 2018 | Teixeira AL |
| 6 | 0 | 2019 | Ma YL |
| 6 | 0 | 2011 | Naumann M |
| 6 | 0 | 2021 | Lee JY |
| 6 | 0 | 2019 | Chung SJ |
| 6 | 0 | 2020 | Roelofs K |
| 6 | 0 | 2020 | Cilia R |
| 6 | 0 | 2019 | Lenka A |
| 6 | 0 | 2018 | Finsterer J |
| 6 | 0 | 2019 | Kim YE |
| 6 | 0 | 2013 | Gallagher DA |
| 6 | 0 | 2018 | Maetzler W |
| 6 | 0.01 | 2021 | Delorme C |
| 6 | 0 | 2019 | Orngreen MC |
| 6 | 0 | 2013 | Zhang X |
| 6 | 0 | 2016 | Nakamura K |
| 6 | 0 | 2020 | Park JH |
| 6 | 0 | 2021 | Wiesinger GF |
| 6 | 0 | 2018 | Boronat A |
| 6 | 0 | 2013 | Nightingale S |
| 6 | 0 | 2021 | Arora S |
| 6 | 0 | 2016 | Ostrem JL |
| 6 | 0 | 2018 | Martinez-Hernandez E |
| 6 | 0 | 2011 | BARNES TRE |
| 6 | 0 | 2021 | Latorre A |
| 6 | 0 | 2021 | Balestrino R |
| 6 | 0 | 2021 | R Core Team |
| 6 | 0 | 2013 | OLDFIELD RC |
| 6 | 0 | 2021 | Jordbru AA |
| 5 | 0 | 2020 | Sateia MJ |
| 5 | 0.01 | 2019 | Weiss D |
| 5 | 0 | 2020 | Roze E |
| 5 | 0 | 2021 | Termsarasab Pichet |
| 5 | 0 | 2021 | Bertolucci F |
| 5 | 0 | 2020 | Videnovic A |
| 5 | 0 | 2021 | Breiman L |
| 5 | 0 | 2021 | Pick S |
| 5 | 0 | 2021 | Doty RL |
| 5 | 0 | 2020 | Perez DL |
| 5 | 0.01 | 2013 | Plazzi Giuseppe |
| 5 | 0 | 2020 | Youn S |
| 5 | 0 | 2021 | Savitt D |
| 5 | 0 | 2018 | Abbott SM |
| 5 | 0 | 2019 | Respondek G |
| 5 | 0 | 2018 | Einspieler C |
| 5 | 0 | 2021 | Regardt M |
| 5 | 0 | 2013 | Hely MA |
| 5 | 0 | 2019 | Lees AJ |
| 5 | 0 | 2019 | Spielberger CD |
| 5 | 0 | 2018 | Olgiati S |
| 5 | 0 | 2020 | Kranick SM |
| 5 | 0 | 2021 | De Antonio M |
| 5 | 0.01 | 2021 | Shin HW |
| 5 | 0 | 2012 | Jeppesen TD |
| 5 | 0 | 2018 | Martikainen MH |
| 5 | 0 | 2019 | Wang J |
| 5 | 0 | 2018 | Kim SM |
| 5 | 0 | 2019 | Savica R |
| 5 | 0 | 2020 | Vuilleumier P |
| 5 | 0 | 2021 | Del Din S |
| 5 | 0 | 2021 | Rusz J |
| 5 | 0 | 2020 | van der Stouwe AMM |
| 5 | 0 | 2018 | Arino H |
| 5 | 0 | 2013 | Boissard R |
| 5 | 0 | 2021 | Netravathi M |
| 5 | 0 | 2020 | Gan-Or Z |
| 5 | 0 | 2013 | Consens FB |
| 5 | 0 | 2015 | Patel N |
| 5 | 0 | 2019 | Brazete JR |
| 5 | 0 | 2018 | Esposito G |
| 5 | 0 | 2018 | DRESSLER D |
| 5 | 0 | 2019 | Earhart GM |
| 5 | 0 | 2018 | Garcia-Cazorla A |
| 5 | 0.01 | 2013 | Schuurman PR |
| 5 | 0 | 2017 | de Hemptinne C |
| 5 | 0 | 2020 | Hassan A |
| 5 | 0 | 2018 | Geis C |
| 5 | 0 | 2016 | Ferraye MU |
| 5 | 0 | 2017 | Yamamoto T |
| 5 | 0 | 2015 | Foltynie T |
| 5 | 0 | 2020 | Seppi K |
| 5 | 0 | 2018 | Segawa M |
| 5 | 0 | 2021 | Williams DR |
| 5 | 0 | 2019 | Pedrosa DJ |
| 5 | 0 | 2017 | Winkelmann J |
| 5 | 0 | 2020 | Teodoro T |
| 5 | 0 | 2021 | Mattar MA |
| 5 | 0 | 2018 | van Sonderen A |
| 5 | 0 | 2020 | Pons R |
| 5 | 0 | 2018 | Hall DA |
| 5 | 0 | 2018 | Hayflick SJ |
| 5 | 0 | 2018 | OToole O |
| 5 | 0 | 2013 | UCHIYAMA M |
| 5 | 0 | 2019 | Hopfner F |
| 5 | 0 | 2021 | Zong M |
| 5 | 0 | 2013 | Brooks PL |
| 5 | 0 | 2016 | Mazzone P |
| 5 | 0 | 2019 | Troster AI |
| 5 | 0 | 2019 | Kumar A |
| 5 | 0 | 2021 | Mahajan A |
| 5 | 0 | 2020 | Jimenez-Jimenez FJ |
| 5 | 0 | 2018 | LECKMAN JF |
| 5 | 0 | 2013 | Gagnon Jean-Francois |
| 5 | 0 | 2021 | Patterson MC |
| 5 | 0 | 2013 | Knudsen S |
| 5 | 0 | 2016 | Lee CW |
| 5 | 0 | 2021 | Rider LG |
| 5 | 0 | 2021 | Martens KAE |
| 5 | 0 | 2016 | FOA EB |
| 5 | 0 | 2019 | Waln O |
| 5 | 0 | 2021 | Carson A |
| 5 | 0 | 2016 | Goldman JG |
| 5 | 0 | 2018 | Puschmann A |
| 5 | 0 | 2021 | de Oliveira DS |
| 5 | 0 | 2013 | Lanfranchi PA |
| 5 | 0 | 2018 | Saunders-Pullman R |
| 5 | 0 | 2020 | Tabrizi SJ |
| 5 | 0 | 2013 | Zucconi M |
| 5 | 0 | 2017 | Walker Z |
| 5 | 0 | 2010 | Miller JM |
| 5 | 0 | 2013 | Zhang JH |
| 5 | 0 | 2018 | Planaguma J |
| 5 | 0 | 2021 | Kim J |
| 5 | 0 | 2021 | Dickson DW |
| 5 | 0 | 2018 | Kruer MC |
| 5 | 0 | 2021 | Zhang J |
| 5 | 0 | 2021 | Boucetta S |
| 5 | 0 | 2019 | Chang FCF |
| 5 | 0 | 2019 | Zhang L |
| 5 | 0 | 2021 | Defazio G |
| 5 | 0 | 2021 | de Souza JM |
| 5 | 0 | 2019 | HARDING AE |
| 5 | 0 | 2021 | Tiffreau V |
| 5 | 0 | 2020 | Nicholson TR |
| 5 | 0 | 2021 | Bargiotas P |
| 5 | 0 | 2021 | Horn A |
| 5 | 0 | 2021 | Wu T |
| 5 | 0 | 2019 | Meles SK |
| 5 | 0 | 2018 | Mestre TA |
| 5 | 0 | 2019 | Ludwig L |
| 5 | 0 | 2018 | Teitelbaum P |
| 4 | 0 | 2014 | Morrison S |
| 4 | 0 | 2010 | Hening WA |
| 4 | 0 | 2012 | Miyasaki JM |
| 4 | 0 | 2011 | OBrien LM |
| 4 | 0 | 2019 | Berman BD |
| 4 | 0 | 2010 | Jain S |
| 4 | 0 | 2021 | Begue I |
| 4 | 0 | 2021 | Ananth AL |
| 4 | 0 | 2015 | Fujishiro H |
| 4 | 0 | 2020 | Gasca-Salas C |
| 4 | 0 | 2017 | Saitsu H |
| 4 | 0 | 2013 | Billiard M |
| 4 | 0 | 2021 | Boehler JF |
| 4 | 0 | 2017 | Provini F |
| 4 | 0 | 2019 | Barow E |
| 4 | 0 | 2015 | Schrock LE |
| 4 | 0 | 2020 | Lubomski M |
| 4 | 0 | 2011 | Tenback DE |
| 4 | 0 | 2016 | Yoritaka A |
| 4 | 0 | 2021 | Aggarwal A |
| 4 | 0 | 2012 | Scott BL |
| 4 | 0 | 2015 | Postuma Ronald B |
| 4 | 0.01 | 2015 | Woerner MG |
| 4 | 0 | 2011 | van Harten PN |
| 4 | 0 | 2016 | Trillenberg P |
| 4 | 0 | 2020 | BAGBY RM |
| 4 | 0 | 2015 | Freed CR |
| 4 | 0 | 2017 | Marshall VL |
| 4 | 0 | 2020 | Liu Y |
| 4 | 0 | 2019 | 2014 |
| 4 | 0 | 2012 | Huang CR |
| 4 | 0 | 2021 | Aithal S |
| 4 | 0 | 2016 | Vitek JL |
| 4 | 0 | 2013 | Lin FC |
| 4 | 0 | 2010 | Gann H |
| 4 | 0 | 2012 | Taivassalo T |
| 4 | 0 | 2019 | Cerasa A |
| 4 | 0 | 2017 | Harper P |
| 4 | 0 | 2013 | Gironell A |
| 4 | 0 | 2020 | Kierkegaard M |
| 4 | 0 | 2013 | Mathis J |
| 4 | 0 | 2017 | Pennestri MH |
| 4 | 0 | 2012 | Halliday DM |
| 4 | 0 | 2016 | Fox MD |
| 4 | 0 | 2019 | Christensen JAE |
| 4 | 0 | 2012 | Kulisevsky J |
| 4 | 0 | 2021 | Bentivoglio AR |
| 4 | 0 | 2011 | Smith Y |
| 4 | 0 | 2017 | Koo BB |
| 4 | 0 | 2015 | Kagi G |
| 4 | 0 | 2019 | Abadi M |
| 4 | 0 | 2011 | Pappa S |
| 4 | 0 | 2011 | KOLLER W |
| 4 | 0 | 2013 | Cornelius JR |
| 4 | 0 | 2019 | Ali F |
| 4 | 0 | 2021 | Allen NM |
| 4 | 0 | 2012 | Shill H |
| 4 | 0 | 2016 | Schrader C |
| 4 | 0 | 2017 | Sirigu A |
| 4 | 0 | 2017 | Swedo SE |
| 4 | 0 | 2013 | HENDERSON JM |
| 4 | 0 | 2013 | Cortese S |
| 4 | 0 | 2016 | Fekete R |
| 4 | 0 | 2013 | Fronczek R |
| 4 | 0 | 2010 | Cooper IS |
| 4 | 0 | 2020 | de Lange FP |
| 4 | 0 | 2012 | ZIGMOND AS |
| 4 | 0 | 2020 | Leen WG |
| 4 | 0 | 2013 | Federlein J |
| 4 | 0 | 2010 | Kish SJ |
| 4 | 0 | 2020 | LaFrance WC |
| 4 | 0 | 2021 | Bernstein DP |
| 4 | 0 | 2016 | Wang JL |
| 4 | 0 | 2017 | Guilleminault C |
| 4 | 0 | 2019 | APA |
| 4 | 0 | 2016 | Abdo WF |
| 4 | 0 | 2020 | HAUBENBERGER D |
| 4 | 0 | 2020 | Kaasinen V |
| 4 | 0 | 2017 | Kim YK |
| 4 | 0 | 2020 | Bologna M |
| 4 | 0 | 2013 | LANDIS JR |
| 4 | 0 | 2013 | Gomez-Choco MJ |
| 4 | 0 | 2013 | Ju YE |
| 4 | 0 | 2016 | Damier P |
| 4 | 0 | 2015 | Kurian MA |
| 4 | 0 | 2019 | BROOK JD |
| 4 | 0 | 2010 | Mahone EM |
| 4 | 0 | 2013 | Franceschini C |
| 4 | 0 | 2011 | Lieberman JA |
| 4 | 0 | 2020 | Castiglioni C |
| 4 | 0 | 2013 | Burns JW |
| 4 | 0 | 2020 | Head RA |
| 4 | 0 | 2012 | Lencer R |
| 4 | 0 | 2014 | McAuley JH |
| 4 | 0 | 2015 | Frucht SJ |
| 4 | 0 | 2010 | Schoemaker MM |
| 4 | 0 | 2015 | Burkhard Pierre R |
| 4 | 0 | 2013 | JACOBS DJ |
| 4 | 0 | 2013 | Kane JM |
| 4 | 0 | 2012 | Galvan A |
| 4 | 0 | 2020 | Li JY |
| 4 | 0 | 2010 | Montgomery EB |
| 4 | 0.01 | 2013 | Cochen V |
| 4 | 0 | 2014 | Jahanshahi M |
| 4 | 0 | 2017 | Kulkarni N |
| 4 | 0 | 2021 | Beach TG |
| 4 | 0 | 2014 | Dawson G |
| 4 | 0 | 2016 | Udd B |
| 4 | 0 | 2013 | Cicolin A |
| 4 | 0 | 2020 | IKEDA K |
| 4 | 0 | 2014 | Flora ED |
| 4 | 0 | 2020 | Jenner P |
| 4 | 0 | 2010 | Stefansson H |
| 4 | 0 | 2013 | Cygan F |
| 4 | 0 | 2013 | HENLEY K |
| 4 | 0 | 2015 | Cif L |
| 4 | 0 | 2013 | Lavault S |
| 4 | 0 | 2016 | Garcia-Lorenzo D |
| 4 | 0 | 2014 | Elahi B |
| 4 | 0 | 2010 | DEMIRKIRAN M |
| 4 | 0 | 2016 | Haack TB |
| 4 | 0 | 2016 | CHIU HFK |
| 4 | 0 | 2020 | Cummings JL |
| 4 | 0 | 2021 | Bhat AN |
| 4 | 0 | 2011 | Brin MF |
| 4 | 0 | 2020 | Lee J |
| 4 | 0 | 2020 | Lee H |
| 4 | 0 | 2020 | Campuzano V |
| 4 | 0 | 2013 | Lam SP |
| 4 | 0 | 2013 | Montagna P |
| 4 | 0 | 2013 | CHASE MH |
| 4 | 0 | 2019 | Chen WJ |
| 4 | 0 | 2020 | Hausdorff JM |
| 4 | 0 | 2015 | Shoulson I |
| 4 | 0 | 2013 | HENDRICKS JC |
| 4 | 0 | 2016 | Plaha P |
| 4 | 0 | 2020 | Jacob AE |
| 4 | 0 | 2017 | Contarino MF |
| 4 | 0 | 2019 | Choi KD |
| 4 | 0 | 2017 | Kordower JH |
| 4 | 0 | 2019 | Bostan AC |
| 4 | 0 | 2013 | Miwa H |
| 4 | 0 | 2010 | BAIN PG |
| 4 | 0 | 2017 | Frank MJ |
| 4 | 0 | 2020 | Alcalay RN |
| 4 | 0 | 2013 | Milanov I |
| 4 | 0 | 2013 | Desmurget M |
| 4 | 0 | 2020 | Benninger DH |
| 4 | 0 | 2020 | **WorldMedicalAssociation |
| 4 | 0 | 2017 | Mankodi A |
| 4 | 0 | 2013 | Jicha GA |
| 4 | 0 | 2015 | Lipsman N |
| 4 | 0 | 2016 | Pedroso JL |
| 4 | 0 | 2016 | Zhuchenko O |
| 4 | 0 | 2013 | Molano J |
| 4 | 0 | 2020 | MACDONALD ME |
| 4 | 0 | 2014 | Morton SM |
| 4 | 0.01 | 2013 | Kimura K |
| 4 | 0 | 2020 | Lefaucheur JP |
| 4 | 0 | 2014 | Rosin B |
| 4 | 0 | 2021 | Artusi CA |
| 3 | 0 | 2011 | Crimlisk HL |
| 3 | 0 | 2011 | Brown RJ |
| 3 | 0 | 2011 | Cervera R |
| 3 | 0 | 2010 | Castellanos FX |
| 3 | 0 | 2016 | Andrews C |
| 3 | 0 | 2012 | Cools R |
| 3 | 0 | 2010 | Baron-Cohen S |
| 3 | 0 | 2014 | Hutton SB |
| 3 | 0 | 2016 | Alves G |
| 3 | 0 | 2012 | Aberg K |
| 3 | 0 | 2010 | Limousin P |
| 3 | 0 | 2015 | Barbe MT |
| 3 | 0 | 2010 | Gaenslen A |
| 3 | 0 | 2015 | Buckner RL |
| 3 | 0.02 | 2014 | Helmchen C |
| 3 | 0.03 | 2014 | Gelb DJ |
| 3 | 0 | 2011 | CARLSSON A |
| 3 | 0 | 2010 | Enoka RM |
| 3 | 0 | 2012 | Andreasen NC |
| 3 | 0 | 2010 | Gracies JM |
| 3 | 0 | 2016 | Amtage F |
| 3 | 0 | 2012 | Cho ZH |
| 3 | 0 | 2012 | Alkelai A |
| 3 | 0 | 2014 | Marsden JF |
| 3 | 0 | 2014 | Beuter A |
| 3 | 0 | 2015 | Bryant RA |
| 3 | 0 | 2014 | *AM AC SLEEP MED |
| 3 | 0 | 2010 | Hening W |
| 3 | 0 | 2014 | KLOCKGETHER T |
| 3 | 0 | 2014 | Abbruzzese G |
| 3 | 0 | 2012 | Esposito M |
| 3 | 0 | 2014 | Lynch DR |
| 3 | 0 | 2014 | Doepp F |
| 3 | 0 | 2010 | COHEN LG |
| 3 | 0 | 2015 | Ashburner J |
| 3 | 0 | 2015 | Castrioto A |
| 3 | 0 | 2014 | Ettinger U |
| 3 | 0 | 2011 | Egan MF |
| 3 | 0 | 2014 | Minshew NJ |
| 3 | 0 | 2011 | BONNET M |
| 3 | 0 | 2010 | Hariz MI |
| 3 | 0 | 2014 | COLLEWIJN H |
| 3 | 0 | 2012 | Barone P |
| 3 | 0 | 2011 | Florance NR |
| 3 | 0 | 2012 | Al Hadithy AF |
| 3 | 0.01 | 2014 | HIKOSAKA O |
| 3 | 0 | 2014 | KAPOULA Z |
| 3 | 0 | 2014 | Li H |
| 3 | 0 | 2014 | Balliet R |
| 3 | 0 | 2012 | Chang MH |
| 3 | 0 | 2016 | Anang JBM |
| 3 | 0 | 2012 | AASM |
| 3 | 0 | 2010 | Behnke S |
| 3 | 0 | 2010 | HASSLER R |
| 3 | 0.01 | 2014 | Konczak J |
| 3 | 0 | 2010 | Lam KSL |
| 3 | 0 | 2014 | Hariz GM |
| 3 | 0 | 2010 | Lou JS |
| 3 | 0 | 2014 | Kuhn AA |
| 3 | 0 | 2012 | Bakker PR |
| 3 | 0 | 2012 | Dugger BN |
| 3 | 0 | 2012 | Bressman SB |
| 3 | 0 | 2012 | Argyelan M |
| 3 | 0 | 2010 | Kralic JE |
| 3 | 0 | 2010 | Coubes P |
| 3 | 0 | 2012 | Fabbrini G |
| 3 | 0 | 2014 | Marsden J |
| 3 | 0 | 2010 | Hagell P |
| 3 | 0 | 2012 | Aldrich MS |
| 3 | 0 | 2010 | BECKER G |
| 3 | 0 | 2010 | Leekam S |
| 3 | 0 | 2012 | Dujardin K |
| 3 | 0 | 2015 | Ackermans L |
| 3 | 0 | 2011 | Binzer M |
| 3 | 0 | 2012 | Eckert T |
| 3 | 0 | 2010 | Hornyak M |
| 3 | 0 | 2011 | FINDLEY LJ |
| 3 | 0 | 2011 | Crabtree VM |
| 3 | 0 | 2010 | Geyer HL |
| 3 | 0 | 2014 | BARKER AT |
| 3 | 0 | 2011 | Giladi N |
| 3 | 0 | 2015 | Barton B |
| 3 | 0 | 2014 | Day JW |
| 3 | 0 | 2011 | Glazer WM |
| 3 | 0 | 2011 | Dean CE |
| 3 | 0 | 2015 | Capelle HH |
| 3 | 0 | 2011 | Brashear A |
| 3 | 0 | 2015 | Cosentino C |
| 3 | 0 | 2011 | Happe S |
| 3 | 0 | 2010 | Harris KM |
