# Supplementary material for: Research trends and frontiers in exercise for movement disorders: A bibliometric analysis of global research from 2010 to 2021
Source: Front Aging Neurosci. 2022 Sep 7;14:977100. doi: 10.3389/fnagi.2022.977100 (PMC9491729; doi:10.3389/fnagi.2022.977100)
Supplement: Supplementary file 3 [file Table_2.docx]

**Supplementary Table 2** Summary of citation frequency and centrality for keywords related to exercise for movement disorders.

| **Co-citation frequency** | **Centrality** | **Year** | **Keyword** |
| --- | --- | --- | --- |
| 557 | 0.03 | 2010 | parkinsons disease |
| 355 | 0.05 | 2010 | movement disorder |
| 166 | 0.04 | 2010 | deep brain stimulation |
| 156 | 0.03 | 2010 | diagnosis |
| 145 | 0.03 | 2010 | rem sleep |
| 142 | 0.03 | 2010 | disease |
| 140 | 0.04 | 2010 | essential tremor |
| 129 | 0.04 | 2010 | children |
| 97 | 0.03 | 2010 | dementia |
| 96 | 0.03 | 2010 | prevalence |
| 96 | 0.04 | 2010 | quality of life |
| 93 | 0.04 | 2010 | dystonia |
| 92 | 0.02 | 2011 | symptom |
| 89 | 0.02 | 2011 | rem sleep behavior disorder |
| 89 | 0.05 | 2010 | restless legs syndrome |
| 85 | 0.03 | 2010 | mutation |
| 84 | 0.04 | 2010 | basal ganglia |
| 79 | 0.03 | 2010 | double blind |
| 78 | 0.02 | 2010 | reliability |
| 72 | 0.05 | 2010 | risk |
| 66 | 0.02 | 2011 | scale |
| 65 | 0.05 | 2010 | huntingtons disease |
| 65 | 0.03 | 2010 | parkinson disease |
| 62 | 0.02 | 2011 | neurodegenerative disease |
| 61 | 0.03 | 2010 | subthalamic nucleus |
| 60 | 0.04 | 2010 | therapy |
| 58 | 0.06 | 2010 | performance |
| 55 | 0.04 | 2010 | association |
| 54 | 0.02 | 2010 | progressive supranuclear palsy |
| 54 | 0.02 | 2011 | rating scale |
| 52 | 0.03 | 2011 | adult |
| 51 | 0.05 | 2010 | impairment |
| 49 | 0.02 | 2010 | multiple system atrophy |
| 49 | 0.01 | 2013 | validity |
| 48 | 0.03 | 2010 | feature |
| 48 | 0.02 | 2014 | functional movement disorder |
| 45 | 0.02 | 2010 | classification |
| 45 | 0.03 | 2010 | tardive dyskinesia |
| 44 | 0.01 | 2012 | management |
| 43 | 0.06 | 2010 | clinical feature |
| 42 | 0.07 | 2010 | brain |
| 41 | 0.03 | 2010 | alzheimers disease |
| 41 | 0.01 | 2012 | mild cognitive impairment |
| 40 | 0.02 | 2013 | cerebral palsy |
| 40 | 0.02 | 2010 | conversion disorder |
| 39 | 0.04 | 2011 | dysfunction |
| 39 | 0.04 | 2011 | risk factor |
| 39 | 0.01 | 2012 | tremor |
| 38 | 0.02 | 2011 | abnormality |
| 38 | 0.03 | 2011 | metaanalysis |
| 38 | 0.03 | 2010 | motor |
| 37 | 0.03 | 2012 | gait |
| 37 | 0.02 | 2011 | model |
| 36 | 0.03 | 2010 | depression |
| 36 | 0.02 | 2010 | eye movement |
| 36 | 0.02 | 2010 | validation |
| 35 | 0.01 | 2014 | cerebellar ataxia |
| 34 | 0.02 | 2013 | adolescent |
| 34 | 0.04 | 2010 | childhood |
| 33 | 0.03 | 2010 | autism spectrum disorder |
| 33 | 0.04 | 2010 | gene |
| 31 | 0.04 | 2010 | follow up |
| 31 | 0.02 | 2012 | lewy body |
| 30 | 0.01 | 2010 | system |
| 28 | 0.02 | 2010 | differential diagnosis |
| 28 | 0.02 | 2010 | expression |
| 28 | 0.02 | 2012 | protein |
| 27 | 0.02 | 2011 | behavior |
| 27 | 0.03 | 2012 | efficacy |
| 27 | 0.01 | 2010 | individual |
| 27 | 0.01 | 2013 | substantia nigra |
| 26 | 0.02 | 2010 | cervical dystonia |
| 26 | 0.01 | 2010 | multiple sclerosis |
| 26 | 0.01 | 2017 | neurodegeneration |
| 26 | 0.03 | 2011 | pathophysiology |
| 25 | 0.01 | 2013 | physical activity |
| 25 | 0.02 | 2010 | tourette syndrome |
| 24 | 0.04 | 2010 | mechanism |
| 24 | 0.01 | 2011 | randomized controlled trial |
| 23 | 0.02 | 2011 | accuracy |
| 23 | 0.05 | 2011 | activation |
| 23 | 0.01 | 2010 | attention |
| 23 | 0.01 | 2010 | botulinum toxin |
| 23 | 0.02 | 2011 | exercise |
| 23 | 0.01 | 2016 | impact |
| 23 | 0.01 | 2014 | polymyositi |
| 23 | 0 | 2010 | psychogenic movement disorder |
| 23 | 0 | 2011 | spinocerebellar ataxia |
| 22 | 0.01 | 2015 | diagnostic criteria |
| 22 | 0.01 | 2014 | network |
| 22 | 0.02 | 2013 | pathology |
| 22 | 0.01 | 2010 | questionnaire |
| 21 | 0.01 | 2015 | balance |
| 21 | 0.01 | 2014 | ptsd |
| 21 | 0.01 | 2015 | schizophrenia |
| 21 | 0.01 | 2013 | skeletal muscle |
| 20 | 0.01 | 2017 | anxiety |
| 20 | 0.01 | 2012 | criteria |
| 20 | 0.02 | 2014 | patient |
| 20 | 0.01 | 2014 | rehabilitation |
| 19 | 0.02 | 2010 | deficiency |
| 19 | 0.01 | 2010 | deficit |
| 19 | 0.01 | 2010 | globus pallidus |
| 19 | 0.01 | 2012 | positron emission tomography |
| 19 | 0 | 2011 | trial |
| 18 | 0.01 | 2010 | disability |
| 18 | 0.03 | 2010 | epilepsy |
| 18 | 0.01 | 2010 | myoclonus |
| 18 | 0 | 2016 | myotonic dystrophy |
| 18 | 0.01 | 2016 | posttraumatic stress disorder |
| 18 | 0.03 | 2014 | stimulation |
| 17 | 0.01 | 2014 | dermatomyositi |
| 17 | 0.01 | 2011 | epidemiology |
| 17 | 0.01 | 2010 | experience |
| 17 | 0.02 | 2014 | spectrum |
| 17 | 0.01 | 2010 | subthalamic nucleus stimulation |
| 16 | 0.02 | 2010 | age |
| 16 | 0 | 2011 | chorea |
| 16 | 0.01 | 2011 | parkinsonism |
| 16 | 0.01 | 2010 | pattern |
| 16 | 0 | 2012 | placebo controlled trial |
| 16 | 0 | 2019 | progression |
| 15 | 0 | 2020 | connectivity |
| 15 | 0.01 | 2014 | people |
| 15 | 0 | 2011 | phenotype |
| 15 | 0 | 2020 | s disease |
| 14 | 0.02 | 2010 | attention deficit/hyperactivity disorder |
| 14 | 0.01 | 2011 | brain stem |
| 14 | 0.01 | 2012 | cell |
| 14 | 0 | 2012 | coordination |
| 14 | 0 | 2016 | de novo mutation |
| 14 | 0 | 2018 | intervention |
| 14 | 0.01 | 2011 | oxidative stress |
| 14 | 0.01 | 2015 | psychotherapy |
| 14 | 0.01 | 2012 | safety |
| 14 | 0.01 | 2013 | transcranial magnetic stimulation |
| 14 | 0.01 | 2014 | trauma |
| 13 | 0.01 | 2011 | cognitive behavioral therapy |
| 13 | 0.01 | 2013 | cognitive impairment |
| 13 | 0 | 2012 | early marker |
| 13 | 0 | 2010 | muscle |
| 13 | 0.01 | 2012 | obsessive compulsive disorder |
| 13 | 0.01 | 2011 | strength |
| 13 | 0.01 | 2013 | variability |
| 12 | 0.01 | 2013 | dementia with lewy body |
| 12 | 0.01 | 2010 | disorder |
| 12 | 0.01 | 2013 | disturbance |
| 12 | 0.01 | 2012 | dopamine |
| 12 | 0 | 2012 | emdr |
| 12 | 0.01 | 2016 | identification |
| 12 | 0 | 2018 | in vivo |
| 12 | 0.01 | 2019 | modulation |
| 12 | 0.01 | 2012 | motor control |
| 12 | 0.01 | 2012 | periodic limb movement |
| 12 | 0 | 2011 | population |
| 11 | 0.01 | 2012 | alpha synuclein |
| 11 | 0.01 | 2015 | alternating hemiplegia |
| 11 | 0 | 2012 | animal model |
| 11 | 0 | 2014 | apnea |
| 11 | 0 | 2011 | cerebellum |
| 11 | 0.01 | 2010 | dyskinesia |
| 11 | 0 | 2016 | fall |
| 11 | 0.01 | 2010 | frequency |
| 11 | 0 | 2015 | levodopa induced dyskinesia |
| 11 | 0.01 | 2010 | neuron |
| 11 | 0 | 2013 | onset |
| 11 | 0.01 | 2010 | stroke |
| 10 | 0 | 2015 | care |
| 10 | 0 | 2016 | epileptic encephalopathy |
| 10 | 0.01 | 2011 | extrapyramidal symptom |
| 10 | 0 | 2013 | lewy body disease |
| 10 | 0 | 2019 | marker |
| 10 | 0.01 | 2012 | motor cortex |
| 10 | 0.01 | 2015 | muscle strength |
| 9 | 0 | 2016 | amyotrophic lateral sclerosis |
| 9 | 0 | 2016 | ataxia |
| 9 | 0 | 2010 | cortex |
| 9 | 0 | 2011 | delayed emergence |
| 9 | 0 | 2019 | health |
| 9 | 0 | 2015 | mice |
| 9 | 0 | 2012 | mitochondrial myopathy |
| 9 | 0.01 | 2015 | nmda receptor encephaliti |
| 9 | 0 | 2010 | repetitive behavior |
| 9 | 0 | 2016 | sleep disorder |
| 8 | 0 | 2013 | antidepressant |
| 8 | 0 | 2014 | brain perfusion |
| 8 | 0 | 2017 | event |
| 8 | 0 | 2016 | mri |
| 8 | 0 | 2017 | nonmotor symptom |
| 8 | 0 | 2013 | older adult |
| 8 | 0 | 2016 | screening questionnaire |
| 8 | 0 | 2019 | sleep |
| 8 | 0 | 2017 | sleep behavior disorder |
| 8 | 0 | 2018 | surgery |
| 8 | 0 | 2016 | walking |
| 8 | 0 | 2014 | young children |
| 7 | 0 | 2013 | adhd |
| 7 | 0 | 2015 | basalganglia |
| 7 | 0.01 | 2011 | clinical diagnosis |
| 7 | 0 | 2011 | clinical trial |
| 7 | 0 | 2017 | fatigue |
| 7 | 0 | 2020 | functional neurological disorder |
| 7 | 0 | 2016 | globus pallidus internus |
| 7 | 0 | 2018 | inclusion body myositi |
| 7 | 0 | 2013 | neurodegenerative disorder |
| 7 | 0 | 2018 | oscillation |
| 7 | 0 | 2019 | outcm |
| 7 | 0 | 2014 | parkinsons disease patient |
| 7 | 0.01 | 2010 | term follow up |
| 7 | 0 | 2012 | transcranial sonography |
| 6 | 0.01 | 2013 | atonia |
| 6 | 0 | 2011 | atypical antipsychotics |
| 6 | 0 | 2017 | body |
| 6 | 0 | 2015 | cerebrospinal fluid |
| 6 | 0 | 2018 | circuit |
| 6 | 0 | 2015 | d aspartate receptor |
| 6 | 0 | 2018 | deficit hyperactivity disorder |
| 6 | 0 | 2010 | eeg |
| 6 | 0 | 2010 | electrical stimulation |
| 6 | 0 | 2015 | functional connectivity |
| 6 | 0 | 2010 | hyperkinetic movement disorder |
| 6 | 0 | 2021 | idiopathic inflammatory myopathy |
| 6 | 0.01 | 2018 | inhibition |
| 6 | 0 | 2016 | injury |
| 6 | 0 | 2011 | involuntary movement |
| 6 | 0 | 2011 | involvement |
| 6 | 0 | 2016 | machado joseph disease |
| 6 | 0 | 2018 | magnetic resonance imaging |
| 6 | 0 | 2017 | memory |
| 6 | 0 | 2017 | motion |
| 6 | 0 | 2019 | mouse model |
| 6 | 0 | 2012 | movement |
| 6 | 0.01 | 2011 | neuroleptic malignant syndrome |
| 6 | 0 | 2018 | outcome measure |
| 6 | 0 | 2018 | stiff person syndrome |
| 6 | 0.01 | 2013 | task |
| 5 | 0 | 2016 | acid |
| 5 | 0 | 2015 | amplitude |
| 5 | 0.01 | 2011 | autoantibody |
| 5 | 0 | 2013 | autonomic dysfunction |
| 5 | 0 | 2015 | cohort |
| 5 | 0 | 2014 | dynamics |
| 5 | 0 | 2010 | face |
| 5 | 0 | 2019 | family |
| 5 | 0 | 2020 | field |
| 5 | 0 | 2013 | hallucination |
| 5 | 0 | 2013 | olfactory dysfunction |
| 5 | 0 | 2013 | thalamic stimulation |
| 4 | 0 | 2013 | adaptation |
| 4 | 0 | 2012 | aerobic exercise |
| 4 | 0 | 2010 | autism |
| 4 | 0 | 2010 | brain parenchyma sonography |
| 4 | 0 | 2019 | common |
| 4 | 0 | 2015 | complication |
| 4 | 0.01 | 2014 | crystal structure |
| 4 | 0 | 2021 | dance/movement therapy |
| 4 | 0 | 2021 | expansion |
| 4 | 0 | 2018 | general movement |
| 4 | 0 | 2013 | hyperechogenicity |
| 4 | 0 | 2021 | inflammatory myopathy |
| 4 | 0 | 2013 | information |
| 4 | 0 | 2020 | korean version |
| 4 | 0 | 2016 | lesion |
| 4 | 0 | 2017 | limb movement |
| 4 | 0 | 2012 | major depression |
| 4 | 0 | 2018 | mitochondrial dysfunction |
| 4 | 0 | 2020 | motor symptom |
| 4 | 0 | 2013 | muscle emg amplitude |
| 4 | 0 | 2013 | muscle tone |
| 4 | 0 | 2013 | narcolepsy |
| 4 | 0 | 2018 | opsoclonus myoclonus syndrome |
| 4 | 0 | 2018 | paraneoplastic cerebellar degeneration |
| 4 | 0 | 2013 | periodic leg movement |
| 4 | 0 | 2018 | predictor |
| 4 | 0 | 2018 | progressive encephalomyeliti |
| 4 | 0 | 2019 | psychometric property |
| 4 | 0 | 2013 | rbd |
| 4 | 0 | 2019 | receptor |
| 4 | 0 | 2014 | recognition |
| 4 | 0 | 2021 | resistive home exercise |
| 4 | 0 | 2013 | status dissociatus |
| 4 | 0 | 2013 | supplementary motor area |
| 4 | 0 | 2019 | suppression |
| 4 | 0 | 2011 | sydenhams chorea |
| 4 | 0 | 2019 | synchronization |
| 3 | 0 | 2019 | adjustment |
| 3 | 0 | 2012 | aerobic capacity |
| 3 | 0 | 2016 | akathisia |
| 3 | 0 | 2011 | antipsychotic drug |
| 3 | 0 | 2021 | architecture |
| 3 | 0 | 2019 | autophagy |
| 3 | 0 | 2019 | barthel index |
| 3 | 0 | 2021 | biomarker |
| 3 | 0 | 2017 | bipolar disorder |
| 3 | 0 | 2016 | brain injury |
| 3 | 0 | 2016 | breast cancer |
| 3 | 0 | 2011 | central nervous system |
| 3 | 0 | 2017 | children and adolescent |
| 3 | 0 | 2011 | cognitiveimpairment |
| 3 | 0 | 2018 | controlled trial |
| 3 | 0 | 2017 | ctg repeat |
| 3 | 0 | 2021 | dance movement psychotherapy |
| 3 | 0 | 2017 | datscan |
| 3 | 0 | 2018 | db |
| 3 | 0 | 2019 | deep learning |
| 3 | 0 | 2014 | developmental coordination disorder |
| 3 | 0 | 2011 | dna damage |
| 3 | 0 | 2012 | dopamine agonist |
| 3 | 0 | 2019 | emdr therapy |
| 3 | 0 | 2020 | evolution |
| 3 | 0 | 2012 | excessive daytime sleepiness |
| 3 | 0 | 2015 | executive function |
| 3 | 0 | 2017 | exercise intolerance |
| 3 | 0 | 2013 | exposure |
| 3 | 0 | 2014 | eye |
| 3 | 0 | 2014 | facial expression |
| 3 | 0 | 2010 | finger tremor |
| 3 | 0 | 2018 | fmr1 messenger rna |
| 3 | 0 | 2016 | force |
| 3 | 0 | 2020 | friedreichs ataxia |
| 3 | 0 | 2017 | functional neurosurgery |
| 3 | 0 | 2010 | gain |
| 3 | 0 | 2014 | gaucher disease |
| 3 | 0 | 2012 | genome wide association |
| 3 | 0 | 2019 | glut1 deficiency syndrome |
| 3 | 0 | 2011 | haloperidol |
| 3 | 0 | 2012 | hemifacial spasm |
| 3 | 0 | 2018 | holmes tremor |
| 3 | 0 | 2019 | idiopathic rbd |
| 3 | 0 | 2019 | impulse control disorder |
| 3 | 0 | 2018 | infection |
| 3 | 0 | 2019 | instrument |
| 3 | 0 | 2016 | intellectual disability |
| 3 | 0 | 2019 | irlssg |
| 3 | 0 | 2018 | kinase associated neurodegeneration |
| 3 | 0 | 2019 | kinesigenic dyskinesia |
| 3 | 0 | 2020 | laboratory supported criteria |
| 3 | 0 | 2016 | leg movement |
| 3 | 0 | 2016 | leigh syndrome |
| 3 | 0.01 | 2011 | limbic encephaliti |
| 3 | 0 | 2019 | local field potential |
| 3 | 0 | 2016 | long term |
| 3 | 0 | 2018 | low frequency earthquake |
| 3 | 0 | 2021 | magnetic stimulation |
| 3 | 0 | 2011 | manifestation |
| 3 | 0 | 2018 | mds research criteria |
| 3 | 0 | 2016 | medication |
| 3 | 0 | 2020 | metabolism |
| 3 | 0 | 2015 | mortality |
| 3 | 0 | 2017 | multicenter |
| 3 | 0 | 2021 | muscle activity |
| 3 | 0 | 2013 | muscular dystrophy |
| 3 | 0 | 2020 | myoclonus dystonia |
| 3 | 0 | 2019 | myotonic dystrophy type 1 |
| 3 | 0 | 2017 | natural history |
| 3 | 0 | 2020 | neurology |
| 3 | 0 | 2018 | neuromuscular disease |
| 3 | 0 | 2019 | neuromuscular disorder |
| 3 | 0 | 2013 | neurotoxic lesion |
| 3 | 0 | 2020 | nucleus |
| 3 | 0 | 2020 | oculomotor abnormality |
| 3 | 0 | 2018 | organization |
| 3 | 0 | 2013 | parasomnia |
| 3 | 0 | 2014 | paroxysmal dyskinesia |
| 3 | 0 | 2015 | pd |
| 3 | 0 | 2017 | perception |
| 3 | 0 | 2012 | periodic limb movement disorder |
| 3 | 0 | 2017 | phenomenology |
| 3 | 0 | 2011 | physical exercise |
| 3 | 0 | 2011 | physiological tremor |
| 3 | 0 | 2018 | pilot |
| 3 | 0 | 2011 | plasticity |
| 3 | 0 | 2017 | post-traumatic stress disorder |
| 3 | 0 | 2013 | posterior parietal cortex |
| 3 | 0 | 2013 | potential |
| 3 | 0 | 2017 | prefrontal cortex |
| 3 | 0 | 2014 | pressure |
| 3 | 0 | 2021 | program |
| 3 | 0 | 2020 | psychogenic motor symptom |
| 3 | 0 | 2013 | psychosis |
| 3 | 0 | 2017 | purkinje cell |
| 3 | 0 | 2014 | quality |
| 3 | 0 | 2016 | recovery |
| 3 | 0 | 2013 | rem sleep behaviordisorder |
| 3 | 0 | 2020 | repeat |
| 3 | 0 | 2012 | resistance training |
| 3 | 0 | 2012 | restless legssyndrome |
| 3 | 0 | 2021 | rtm |
| 3 | 0 | 2013 | screening tool |
| 3 | 0 | 2020 | seizure |
| 3 | 0 | 2016 | sensitivity |
| 3 | 0 | 2020 | sequence |
| 3 | 0 | 2020 | severity |
| 3 | 0 | 2018 | spect |
| 3 | 0 | 2013 | spectral analysis |
| 3 | 0 | 2012 | stability |
| 3 | 0 | 2015 | stimuli |
| 3 | 0 | 2013 | stress |
| 3 | 0 | 2020 | subtype |
| 3 | 0 | 2020 | subunit |
| 3 | 0 | 2021 | systematic review |
| 3 | 0 | 2013 | target |
| 3 | 0 | 2015 | thalamotomy |
| 3 | 0 | 2014 | tourettes syndrome |
| 3 | 0 | 2021 | transcranial magneticstimulation |
| 3 | 0 | 2010 | upper limb |
| 3 | 0 | 2016 | version |
| 3 | 0 | 2020 | visual search |
| 3 | 0 | 2020 | vitamin e deficiency |
| 3 | 0 | 2021 | wearable sensor |
| 3 | 0 | 2011 | wilsons disease |
| 3 | 0 | 2013 | working memory |
| 3 | 0 | 2012 | writers cramp |
| 2 | 0 | 2014 | 1st episode |
| 2 | 0 | 2010 | 3 elderly population |
| 2 | 0 | 2010 | 40 hz |
| 2 | 0 | 2021 | acquired hepatocerebral degeneration |
| 2 | 0 | 2017 | adcy5 mutation |
| 2 | 0 | 2021 | adenylyl cyclase |
| 2 | 0 | 2016 | advanced parkinsons disease |
| 2 | 0 | 2015 | adverse event |
| 2 | 0 | 2011 | american academy |
| 2 | 0 | 2017 | amygdala |
| 2 | 0 | 2017 | anorexia |
| 2 | 0 | 2015 | anterior insula |
| 2 | 0 | 2016 | antibody |
| 2 | 0 | 2013 | area |
| 2 | 0 | 2013 | arenal volcano |
| 2 | 0 | 2011 | arm |
| 2 | 0 | 2015 | ataxia telangiectasia |
| 2 | 0 | 2014 | atm |
| 2 | 0 | 2013 | attention-deficit hyperactivity disorder |
| 2 | 0 | 2015 | autoimmune encephaliti |
| 2 | 0 | 2013 | avoidance |
| 2 | 0 | 2014 | beta oscillation |
| 2 | 0 | 2014 | bias |
| 2 | 0 | 2014 | biological motion |
| 2 | 0 | 2012 | blepharospasm |
| 2 | 0 | 2011 | blind |
| 2 | 0 | 2015 | blood flow |
| 2 | 0 | 2011 | blood pressure |
| 2 | 0 | 2014 | body movement |
| 2 | 0 | 2012 | body rocking |
| 2 | 0 | 2012 | brain network |
| 2 | 0 | 2013 | c fos expression |
| 2 | 0 | 2016 | case series |
| 2 | 0 | 2013 | cataplexy |
| 2 | 0 | 2016 | caudal fastigial nucleus |
| 2 | 0 | 2013 | central pontine myelinolysis |
| 2 | 0 | 2014 | centronuclear myopathy |
| 2 | 0 | 2012 | cerebellar |
| 2 | 0 | 2011 | cerebellar degeneration |
| 2 | 0 | 2012 | cerebral blood flow |
| 2 | 0 | 2014 | channel |
| 2 | 0 | 2014 | choreoathetosis |
| 2 | 0 | 2011 | clinical analysis |
| 2 | 0 | 2013 | cognitive control |
| 2 | 0 | 2011 | cognitive function |
| 2 | 0 | 2014 | community |
| 2 | 0 | 2011 | complexity |
| 2 | 0 | 2011 | consensus statement |
| 2 | 0 | 2012 | contingent negative variation |
| 2 | 0 | 2010 | corticobasal degeneration |
| 2 | 0 | 2010 | cue |
| 2 | 0 | 2012 | curacao extrapyramidal syndrm |
| 2 | 0 | 2015 | daytime sleepiness |
| 2 | 0 | 2010 | dcd |
| 2 | 0 | 2012 | decision making |
| 2 | 0 | 2015 | deep brain stimulation (dbs) |
| 2 | 0 | 2014 | deepbrain stimulation |
| 2 | 0 | 2012 | delay |
| 2 | 0 | 2015 | dentate nucleus |
| 2 | 0 | 2012 | depression scale |
| 2 | 0 | 2015 | dopamine receptor |
| 2 | 0 | 2015 | dopamine transporter |
| 2 | 0 | 2014 | dynamin 2 |
| 2 | 0 | 2012 | eccentric exercise |
| 2 | 0 | 2014 | emotion perception |
| 2 | 0 | 2012 | excitability |
| 2 | 0 | 2010 | glucose |
| 2 | 0 | 2010 | hemichorea |
| 2 | 0 | 2010 | hemichoreoathetosis |
| 2 | 0 | 2010 | hemidystonia |
| 2 | 0 | 2010 | high frequency stimulation |
| 2 | 0 | 2010 | high functioning autism |
| 2 | 0 | 2010 | hyperventilation |
| 2 | 0 | 2010 | idiopathic parkinsons disease |
| 2 | 0 | 2010 | imitation |
